# Activated P2X receptors can up-regulate the expressions of inflammation-related genes via NF-κB pathway in spotted sea bass (*Lateolabrax maculatus*)

**DOI:** 10.3389/fimmu.2023.1181067

**Published:** 2023-05-04

**Authors:** Zhaosheng Sun, Qian Gao, Youchuan Wei, Zhigang Zhou, Yuxi Chen, Chong Xu, Jiaqi Gao, Danjie Liu

**Affiliations:** ^1^ Key Laboratory of Exploration and Utilization of Aquatic Genetic Resources, Ministry of Education, Shanghai Ocean University, Shanghai, China; ^2^ International Research Center for Marine Biosciences, Ministry of Science and Technology, Shanghai Ocean University, Shanghai, China; ^3^ College of Animal Science and Technology, Guangxi University, Nanning, China; ^4^ SinoNorway Fish Gastrointestinal Microbiota Joint Lab, Feed Research Institute, Chinese Academy of Agricultural Sciences, Beijing, China

**Keywords:** P2X receptors, extracellular ATP, innate immunity, NF-κB pathway, *Lateolabrax maculatus*

## Abstract

P2X receptors, including seven subtypes, i.e., P2X1-7, are the ligand-gated ion channels activated by the extracellular ATP playing the critical roles in inflammation and immune response. Even though the immune functions of P2X receptors have been characterized extensively in mammals, their functions in fish remain largely unknown. In this study, four P2X receptor homologues were characterized in spotted sea bass (*Lateolabrax maculatus*), which were named LmP2X2, *LmP2X4*, *LmP2X5*, and *LmP2X7*. Their tissue distributions and expression patterns were then investigated by real-time quantitative PCR (qPCR). Furthermore, their functions in regulating the expressions of inflammation-associated genes and possible signaling pathway were examined by qPCR and luciferase assay. The results showed that they share similar topological structures, conserved genomic organization, and gene synteny with their counterparts in other species previously investigated. And the four P2X receptors were expressed constitutively in the tested tissues. In addition, the expression of each of the four receptor genes was significantly induced by stimulation of *Edwardsiella tarda* and/or pathogen-associated molecular patterns (PAMPs) *in vivo*. Also, in primary head kidney leukocytes of spotted sea bass, *LmP2X2* and *LmP2X5* were induced by using PAMPs and/or ATP. Notably, the expressions of CCL2, IL-8, and TNF-α recognized as the pro-inflammatory cytokines, and of the four apoptosis-related genes, i.e., caspase3, caspase6, caspase7, and P53, were differentially upregulated in the HEK 293T cells with over-expressed *LmP2X2* and/or *LmP2X7* following ATP stimulation. Also, the over-expression of LmP2X4 can upregulate the expressions of IL-8, caspase6, caspase7, and P53, and *LmP2X5* upregulates of IL-8, TNF-α, caspase7, and P53. Then in the present study it was demonstrated that the activation of any one of the four receptors significantly upregulated the activity of NF-κB promoter, suggesting that the activated *LmP2Xs* may regulate the expressions of pro-inflammatory cytokines via the NF-κB pathway. Taken together, the four P2X receptors were identified firstly from fish species in Perciformes, and they participate in innate immune response of spotted sea bass possibly by regulating the expressions of the inflammation-related genes. Our study provides the new evidences for the P2X receptors’ involvement in fish immunity.

## 1 Introduction

Adenosine triphosphate (ATP) is well known as the universal energy currency in cellular metabolism (1). However, there are growing evidences that ATP is released from activated or stressed cells due to inflammation, body injury, and/or apoptosis to perform its immune functions (1–3). Extracellular ATP is a kind of damage-associated molecular patterns (DAMPs), involved in activating the innate immune defense mechanisms, inflammasome activation, phagocytosis, etc. (1, 4, 5). Importantly, these functions are performed by extracellular ATP *via* the activation of P2 receptors on the surface of cell (5, 6). P2 receptors can be classified into two subfamilies, i.e. P2X as ligand-gated ion channels and P2Y being G protein-coupled receptors (1, 7). P2X receptors consist of 7 members (*P2X1-7*), forming either homo- or hetero-trimeric channels (8, 9). All the P2Xs share similar topological structures, including an extracellular (ECL) region, two transmembrane (TM) domains, and both N- and C-terminal regions in the cytosol (7, 10). Differently, although their natural agonist is extracellular ATP, each P2X receptor has a different affinity for ATP from the micromolar to the millimolar levels (7).

In mammals, P2Xs are widely expressed in immune cells, such as macrophages, T cells, and B cells, to participate in various immune responses, including the release of cytokines, apoptosis, NF-κB activation, etc. (1, 4, 5, 11). Among the P2X subtypes, as the essential moderator of both inflammation and immunity, *P2X7* has received the most attention (12), which has been shown to extensively engage in innate and adaptive immunity (6, 12–17). Besides *P2X7*, *P2X2* and *P2X4* were also involved in the release of pro-inflammatory cytokines, T cell activation, and the differentiation of CD4^+^ T cells (4, 7, 18–23). P2X2/3 or P2X3 activation is associated with inflammatory hyperalgesia (24), and *P2X5* is required for *Listeria monocytogenes*-induced inflammasome activation and IL-1β production (25). In summary, A large number of studies have indicated that P2X receptors play important roles in inflammation and immune response in mammals.

Compared to mammals, evidences for the involvement of P2X receptors in fish inflammation and immunity are still limited. To date, six P2X receptors except *P2X6* have been cloned in zebrafish (*Danio rerio*) (26, 27) and some P2X receptors have also been identified in other fish species, such as *P2X7* in gilthead seabream (*Sparus aurata L.*) (28) and ayu (*Plecoglossus altivelis*) (29), and *P2X2*, *P2X4*, and *P2X7* in Japanese flounder (*Paralichthys olivaceus*) (30–32). Similarly, P2X7 of fish was relatively more studied, which was proved to play a critical role in innate immunity (28, 33). For instances, P2X7 of Japanese flounder was involved in innate immune response by moderating the expressions of pro-inflammatory cytokines (30), and P2X7 in ayu macrophages could mediate cell death, phagocytosis, and bacterial killing (29). In addition, P2X2 and P2X4 in Japanese flounder have been proved to take part in the innate immunity (31, 32). To our knowledge, so for there has been no report on the immunological function of P2X5 in fish. Therefore, the immune functions of P2X receptors in fish need to be extensively studied. Our previous study has indicated that ATP can be released from cells through pannexin1, connexin32, and connexin43 channels in inflammatory situation in spotted sea bass (*Lateolabrax maculatus*) (34). Therefore, it is needed to explore further the functions of extracellular ATP in spotted sea bass. In this study, we characterized four P2X receptors in spotted sea bass, which was their first identification in Perciformes. Their tissue distributions and the regulation of expressions by *Edwardsiella tarda* infection and PAMPs stimulation were then investigated. Furthermore, their functions, particularly for P2X5 unnoticed previously in fish, in regulating the expressions of inflammation-associated genes and possible signaling pathway were examined. Undoubtedly, this study will be helpful in enriching and further understanding the innate immune response mediated by extracellular ATP in fish.

## 2 Materials and methods

### 2.1 Experimental fish

Spotted sea bass (100 ± 10 g) were sourced from a fish farm in Hangzhou city, Zhejiang province, China. Fish were farmed in a fish aquaculture system at 26 ± 2 °C and fed with commercial feed of sea bass once a day for more than two weeks before experiments. Healthy fish without any pathological signs were then selected for the experiments.

### 2.2 Cloning of *P2X*s from spotted sea bass

According to previously described methods, the first strand cDNA was obtained and the full-length cDNA sequences were then cloned and verified as the method described previously (35). In details, total RNA was extracted from head kidney and liver of spotted sea bass for gene cloning using the TRIzol reagent (Ambion, USA). The first strand cDNA was reverse-synthesized from total RNA using the Hifair^®^ II 1st Strand cDNA Synthesis Kit (gDNA digester plus) (YEASEN, China). For the 5´- and 3´-RACE, nested PCR was performed with Premix Ex Taq™ Hot Start Mix (TaKaRa, Japan). The complete coding sequences were verified by sequencing the PCR products amplified using one pair of primers located at the 5´- and 3´- untranslated region. All primers were summarized in **Supplementary Table 1**.

### 2.3 Bioinformatics analysis

Programs on NCBI (https://www.ncbi.nlm.nih.gov/) and Expasy (http://www.expasy.org/tools) website were used to analyze nucleotide & protein sequences and the Ensembl (http://www.ensembl.org/) and NCBI genome database were analyzed to infer the genomic organization & syntenic relationships. Phylogenetic tree and multiple sequence alignment were analyzed by the same methods (35) and then the phylogenetic tree was embellished using the iTOL website (https://itol.embl.de/) (36). Concretely, multiple sequence alignments were performed using the ClustalW program and processed using the GeneDoc program. Phylogenetic tree was constructed by the Neighbour-Joining method using the MEGA X program and the tree were bootstrapped for 10,000 replications.

### 2.4 Tissue expression analysis

Following the same method, 8 tissue samples including liver, spleen, head kidney, intestine, skin, gill, muscle, and brain of healthy spotted sea bass were obtained for the preparation of assay templates (35, 37). The first strand cDNA was reverse-synthesized from 5 μg total RNA using Hifair^®^ II 1st Strand cDNA Synthesis SuperMix (gDNA digester plus) (YEASEN, China). The real-time quantitative PCR (qPCR) was performed in triplicate using Hieff^®^ qPCR SYBR Green Master Mix (No Rox) (YEASEN, China), and all qPCR primers were shown in **Supplementary Table 1**. The relative expression levels of target genes were normalized to that of *EF1α*.

### 2.5 Expression analysis after *in vivo* stimulation

For the challenge experiment, fish were i.p. injected with 500 μL Lipopolysaccharides (LPS, 1 mg/mL), Polyinosinic-polycytidylic acid (Poly (I:C), 1 mg/mL), *Edwardsiella tarda* (1×10^5^ CFU/mL) or phosphate buffered saline (PBS, pH 7.4, Gibco). Tissues were sampled at 6, 12, 24, and 48 h after injection, homogenized in 1 mL TRIzol, and reverse transcribed into cDNA for qPCR analysis as described above (37). *E. tarda* was cultured in tryptic soy agar medium or tryptic soy broth medium at 28°C and prepared as previously described (37). LPS and Poly (I:C) were purchased from Sigma-Aldrich (USA).

### 2.6 Expression analysis after *in vitro* stimulation

Primary head kidney leukocytes of healthy spotted sea bass were isolated by a discontinuous 51% Percoll gradient as previously described (38). The leukocytes (1×10^7^/well) were cultured in a 6-well plate (Corning, USA) with DMEM-F12 medium (Gibco, USA) containing 10% fetal bovine serum (FBS, Gibco) and 1% Pen/Strep (Gibco, USA) in a CO_2_ incubator at 28 °C, and then treated with 50 μg/mL peptidoglycan (PGN, Sigma-Aldrich), 100 μg/mL LPS, 50 μg/mL Poly (I:C), 100 μM or 1mM ATP (Sigma-Aldrich, USA), an equal volume of PBS was used as the control. Each condition was done in Quadruplicate. Cell samples were collected for qPCR at 6, 12, 24, and 48 h after stimulation. Total RNA was extracted using TRIzol reagent and reverse transcribed into cDNA for qPCR analysis as described above.

### 2.7 Expression analysis of inflammation-associated genes in *Lm*P2Xs-overexpressing HEK 293T cells after ATP stimulation

Based on the obtained CDS sequences, the recombinant pcDNA3.1 expression plasmid for *Lm*P2Xs were constructed, which were named as pcDNA3.1-*Lm*P2X2, pcDNA3.1-*Lm*P2X4, pcDNA3.1-*Lm*P2X5 and pcDNA3.1-*Lm*P2X7. HEK 293T cells were then transfected with pcDNA3.1-*Lm*P2Xs or pcDNA3.1 using the PEI 40K Transfection Reagent (Servicebio, China) according to the manufacturer’s instruction. The transfected cells were cultured in DMEM basic medium (Gibco, USA) containing 10% FBS and 1% Pen/Strep in a CO_2_ incubator at 37°C and the medium was replaced with fresh medium after 24 h. The cells were then stimulated with different concentrations of ATP (100 μM, 1 mM, or 5 mM), and were collected for qPCR after 6 h. The empty plasmid transfected cells by PBS stimulation served as the negative control. Each condition was done in triplicate. Total RNA was extracted using TRIzol reagent and reverse transcribed into cDNA for qPCR analysis as described above. All qPCR primers were shown in **Supplementary Table 1**.

### 2.8 Luciferase assay

The pcDNA3.1-*Lm*P2Xs plasmid or pcDNA3.1 plasmid was co-transfected with the pGL4.32 (luc2P/NF-κB-RE/Hygro, NF-κB reporter plasmid, Promega) and pRL-TK (renilla luciferase control plasmid, Promega) into HEK 293T cells. The transfected cells were cultured for 24 h, and then were stimulated with ATP (100 μM, 1 mM, or 5 mM) or PBS (control). After 6 h, the cells were collected and then lysed for measuring the luciferase activity by using the dual-luciferase reporter assay system (Promega, USA) on a LumiPro (YPHBIO, China). Each condition was done in triplicate.

### 2.9 Subcellular localization

The pEGFP-N1 expression plasmid containing the coding sequence (CDS) of *Lm*P2X2, *Lm*P2X4, *Lm*P2X5, or *Lm*P2X7 were constructed, i.e. pEGFP-N1-*Lm*P2X2, pEGFP-N1-*Lm*P2X4, pEGFP-N1-*Lm*P2X5, and pEGFP-N1-*Lm*P2X7. We then transfected the recombinant pEGFP-N1 plasmids into HEK 293T cells by the same methods as before. As described by the method described previously (39), the transfected cells were cultured in coverslips pretreated with poly-L-lysine (Solarbio, China) at 24 h. Next, cells were stained with DAPI (Solarbio, China) and observed using a laser confocal microscope (Leica TCS SP8, Germany).

### 2.10 Statistical analysis of date

Data were analyzed using the SPSS package 20.0 by one-way ANOVA, “p<0.05” or “p<0.01” is considered significant between treatment groups and control groups (35).

## 3 Results

### 3.1 Sequence features of the four P2X receptors in spotted sea bass

The sequences obtained in this study have been deposited in the NCBI database with the following accession numbers: OP651016 (*LmP2X2*), OP651019 (*LmP2X4*), OP651020 (*LmP2X5*), and OP651021 (*LmP2X7*). Meanwhile, the sequence features of *Lm*P2Xs, including the length of cDNA, 5´-untranslated region (UTR), 3´-UTR, and open reading frame (ORF) were summarized in **Table 1**. In addition, the cDNA and deduced amino acid sequence information of *Lm*P2Xs was shown in **Supplementary Figure 1**.

**Table 1:** Sequence Characterization of *Lm*P2Xs.

Features	*Lm*P2X2	*Lm*P2X4	*Lm*P2X5	*Lm*P2X7
accession numbers	OP651016	OP651019	OP651020	OP651021
cDNA length (bp)	1880	1352	2228	1994
5′-UTR (bp)	113	25	214	137
3′-UTR (bp)	519	136	808	114
ORF (bp)	1248	1191	1206	1743
Amino acids (aa)	415	396	401	580
PKC site	yes	yes	yes	yes
P2X signature motif	yes	yes	yes	yes
YXXXK motif	yes	yes	yes	yes
LPS/lipid-binding domain	no	no	no	yes

**Figure 1:**
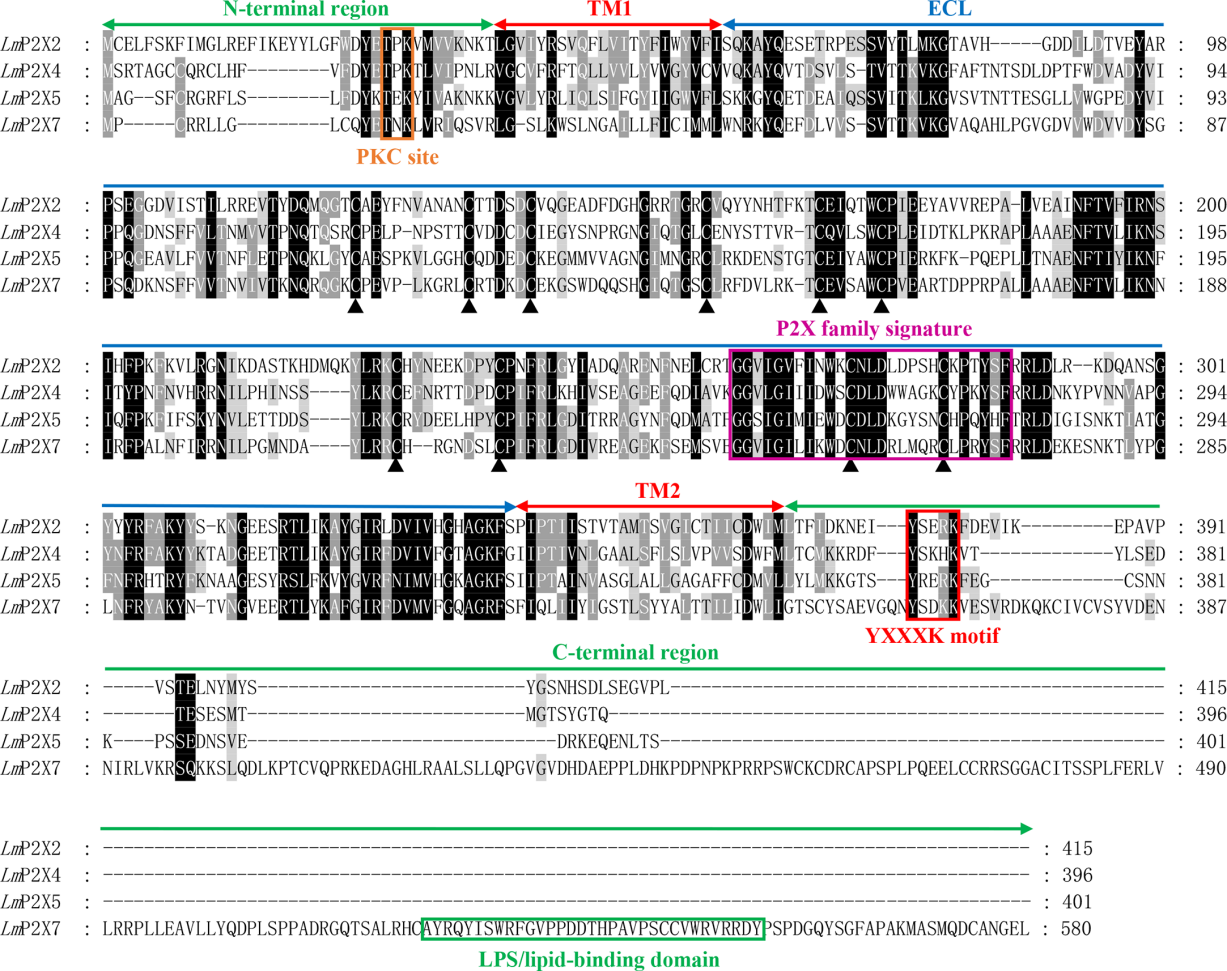
Multiple sequence alignment analysis of P2Xs. The N-terminal region, two transmembrane domains (TM1-2), the extracellular loop (ECL), and the C-terminal region of *Lm*P2Xs are marked above the alignment. Symbol (▴) indicates the conserved cysteine residues. The PKC site, P2X family signature motif, YXXXK motif, and LPS/lipid-binding domain are boxed in orange, pink, red, and green, respectively.

**Figure 2:**
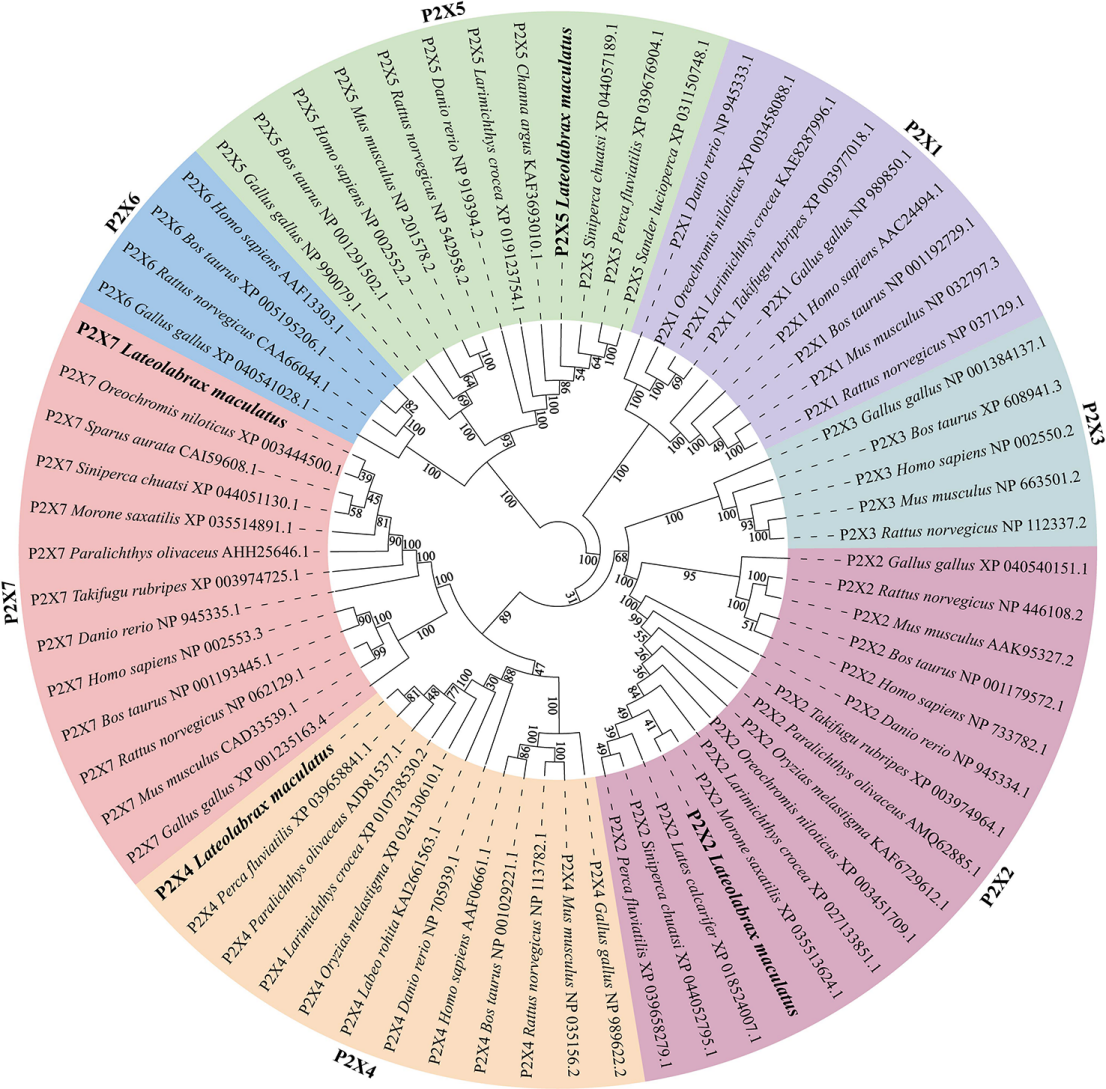
Phylogenetic tree analysis of *Lm*P2Xs with the selected P2X family members. Phylogenetic tree was constructed using the NJ method within the MEGAX program and run for 10,000 replications. The percentages of bootstrap values for branches are indicated and the *Lm*P2Xs are shown in bold.

Like other species, multiple sequence alignment revealed that *Lm*P2Xs retain two highly conserved transmembrane domains (TM1 and TM2), a P2X receptor signature motif, and 10 conserved cysteine residues in the extracellular domain; At the same time, they contain the conserved protein kinase C (PKC) site and the YXXXK motif which located in the intracellular N-terminal region and C-terminal region, respectively (**Figure 1**, **Supplementary Figure 2**). Compared with other *Lm*P2X members, *Lm*P2X7 has a longer C-terminal region and contains an additional LPS/lipid-binding domain (**Figure 1**). In addition, the phylogenetic tree was constructed to infer the homologous relationships among P2Xs from various species. As shown in **Figure 2**, *Lm*P2Xs were clustered with their homologues from other species, well reflecting the phylogeny of the chosen species.

### 3.2 Genomic organization and synteny of the four P2X receptors in spotted sea bass

**Figure 3:**
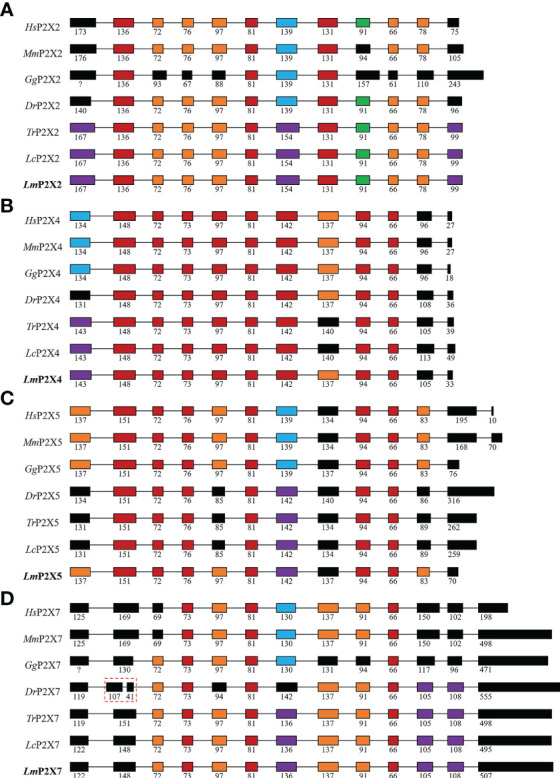
Genomic organization of *P2X2* **(A)**, *P2X4* **(B)**, *P2X5* **(C)**, and *P2X7* **(D)**. The solid boxes indicate coding exon. The numbers indicate the size (bp) of exons. Note that the size of exons and introns is disproportionate.

**Figure 4:**
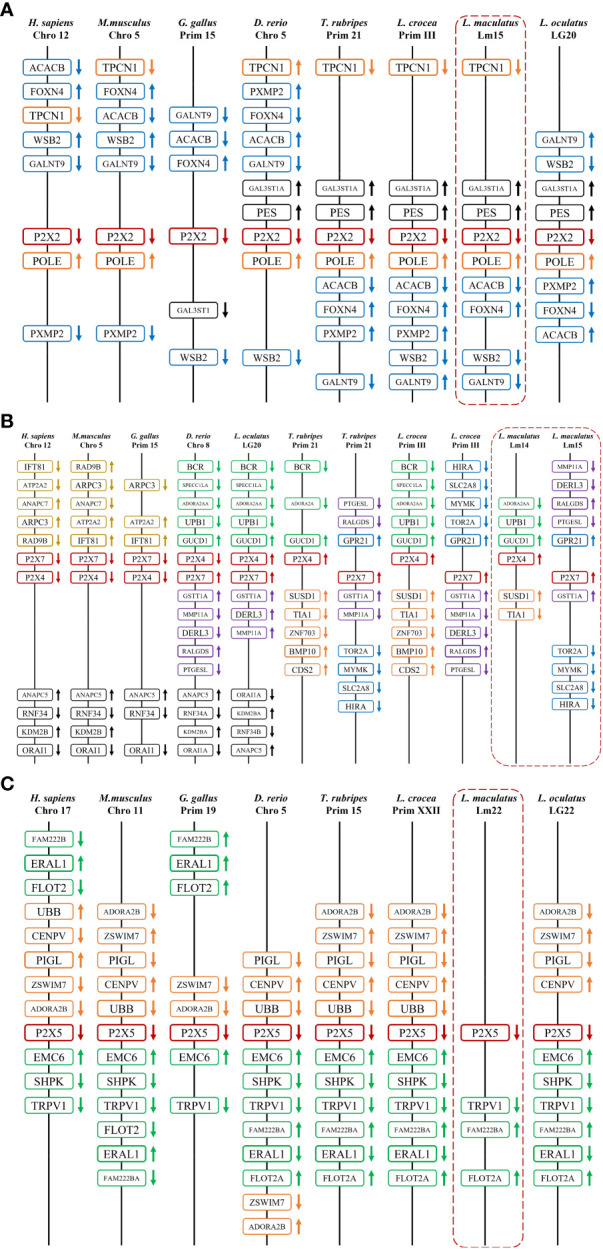
Gene synteny of *P2X2* **(A)**, *P2X4/7* **(B)**, and *P2X5* **(C)**. The *Lm*P2Xs genome sequence data were obtained from the spotted sea bass genome database (https://www.ncbi.nlm.nih.gov/-genome/43909). Synteny information for other vertebrates was retrieved from the Ensembl database (http://www.ensembl.org/index.html). Arrows indicate transcription orientations.

By comparing cDNA sequences with genomic sequences, the genomic organizations of P2Xs were confirmed (**Figure 3**). The CDS region of all *LmP2X*s except *LmP2X7* contained 12 exons and 11 introns, and the size of the exon at the same position was comparable. Corresponding to the longer C-terminal region, *LmP2X7* contains an additional exon in the C-terminal region. Separately, the number and size of exons of *LmP2X*s were highly conserved with their homologous of other species. Overall, the genomic organization of *P2X*s was highly conserved in different species. Furthermore, gene synteny showed that the *P2X2* and *P2X5* locus have been well conserved during evolution, with *P2X2* linked to *FOXN4* and the *P2X5* linked to *TRPV1* (**Figures 4A, C**). On the contrary, the *P2X4* and *P2X7* locus have been relatively unconserved. Specifically, *P2X4* was closely linked to *P2X7* in human, mouse, chicken, zebrafish, and spotted gar, but not in fugu, large yellow croaker and spotted sea bass (**Figure 4B**). However, it is worth noting that *P2X4* was linked to *GUCD1* and *ADORA2AA*, and P2X7 was linked to *GSTT1A* and *MMP11A* in fish (**Figure 4B**).

### 3.3 Tissue distributions of the four P2X receptors in spotted sea bass

**Figure 5:**
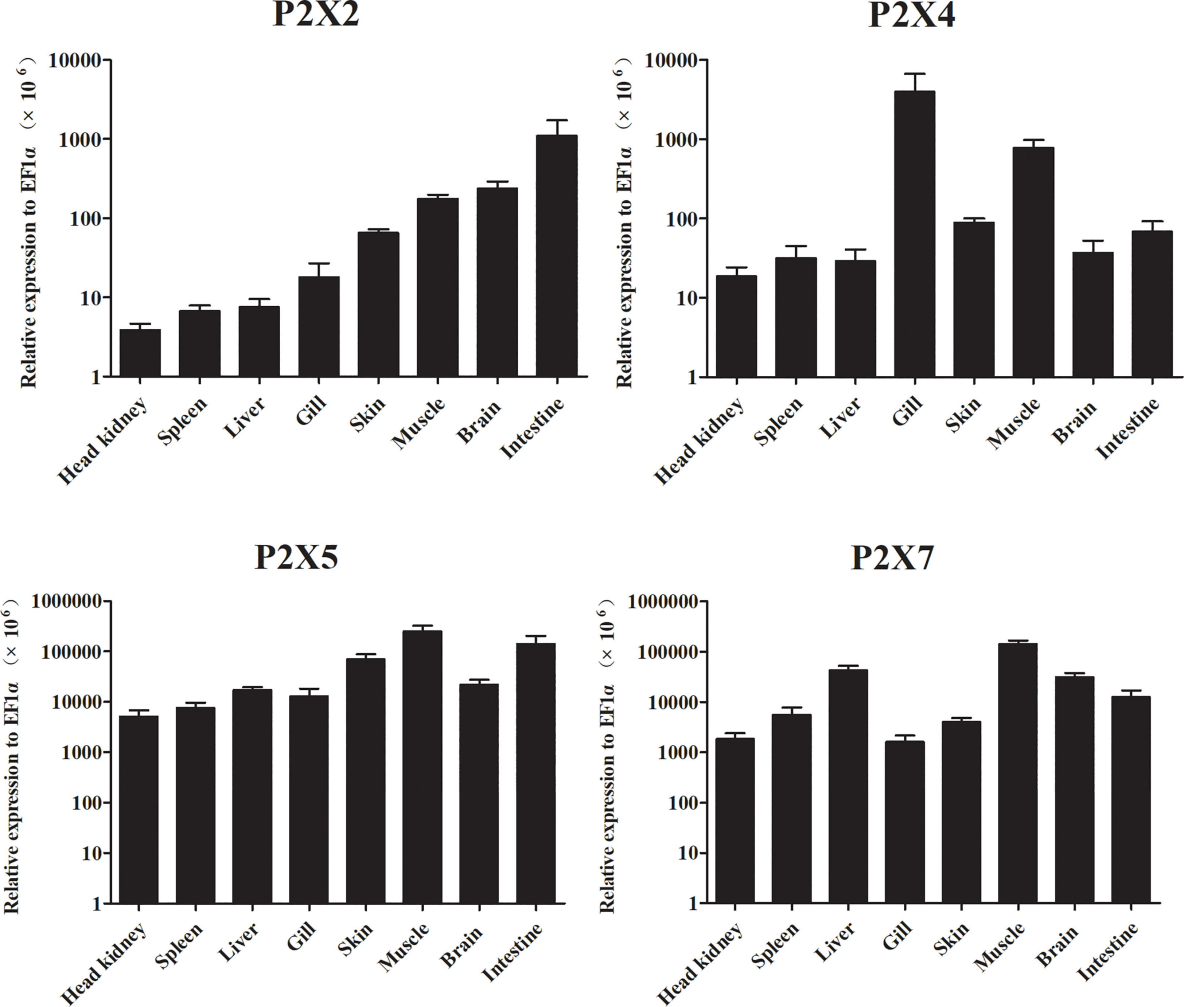
Expression analysis of *Lm*P2Xs in tissues. The expression levels of each gene were normalized to that of EF1α. Data are presented as mean + SEM (N= 4).

Expressions of *LmP2X2*, *LmP2X4*, *LmP2X5*, and *LmP2X7* were analyzed in various tissues by qPCR analysis (**Figure 5**). The results showed that all four *LmP2X*s were constitutively expressed in the tissues examined. The highest expressions of *LmP2X*s were observed in intestine, gill, and muscle, respectively. In addition, their expressions were lower in spleen and head kidney. Interestingly, the highest expression of *LmP2X4* and the lower expressions of other *LmP2X*s were both found in gill. Taken together, the differences in the distribution trends of *LmP2X*s indicated that they were differentially regulated in eight tissues.

### 3.4 Expressions of the four P2X receptors in spotted sea bass following injection with Poly (I:C), LPS, or *Edwardsiella tarda*

**Figure 6:**
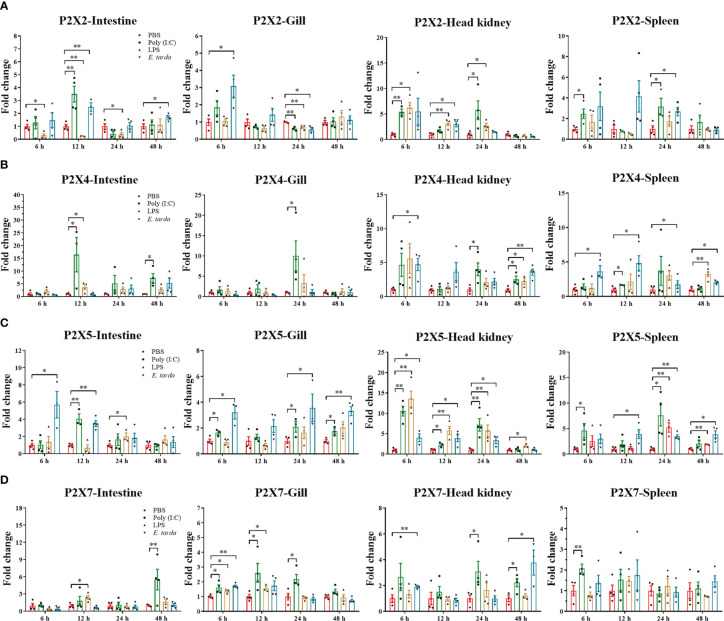
Expressions of *Lm*P2X2 **(A)**, *Lm*P2X4 **(B)**, *Lm*P2X5 **(C)**, and *Lm*P2X7 **(D)** after LPS, Poly (I:C) or *Edwardsiella tarda* challenge. Spotted sea bass was i.p. injected with 500 μL LPS (1 mg/mL), Poly (I:C) (1 mg/mL), *E tarda* (1×10^5^ CFU/mL), or PBS (control) and tissues were sampled at 6, 12, 24 and 48 h for qPCR analysis. The relative expression levels of target genes were normalized to that of EF1α and fold change was obtained by comparing the relative gene expression level of the treated group and control group (defined as 1). Data are shown as mean + SEM (N=3). *P<0.05 and **P<0.01 are considered significant difference.

The mRNA expressions of the four P2X receptors in response to Poly (I:C), LPS, and *E. tarda* challenge were investigated in intestine, head kidney, spleen, and gill (**Figure 6**). In intestine, head kidney, and spleen, the expression of *LmP2X2* was up-regulated to different degrees after Poly (I:C) and *E. tarda* stimulation, but down-regulated in intestine after LPS stimulation; In gill, the expression of *LmP2X2* was down-regulated after three kinds of stimulation at 24 h and only up-regulated at 6 h after *E. tarda* stimulation (**Figure 6A**). As shown in **Figure 6B**, *LmP2X4* were also up-regulated in intestine [after Poly (I:C) and LPS stimulation], gill [only at 24 h after Poly (I:C) stimulation], head kidney, and spleen. Noteworthy, *LmP2X5* was induced in all examined tissues after the three kinds of stimulation, except in gill after LPS stimulation where no significant changes were observed (**Figure 6C**). Like *LmP2X5*, the expression of *LmP2X7* was up-regulated to different degrees in all tissues after Poly (I:C) stimulation, furthermore, LPS and *E. tarda* stimulation also induced *LmP2X7* in head kidney and gill (**Figure 6D**). Collectively, all the genes were up-regulated to different degrees after stimulation, but their expression patterns differed in different tissues.

### 3.5 Expressions of the four P2X receptors in primary head kidney leucocytes of spotted sea bass after PGN, LPS, Poly (I:C), or ATP stimulation

**Figure 7:**
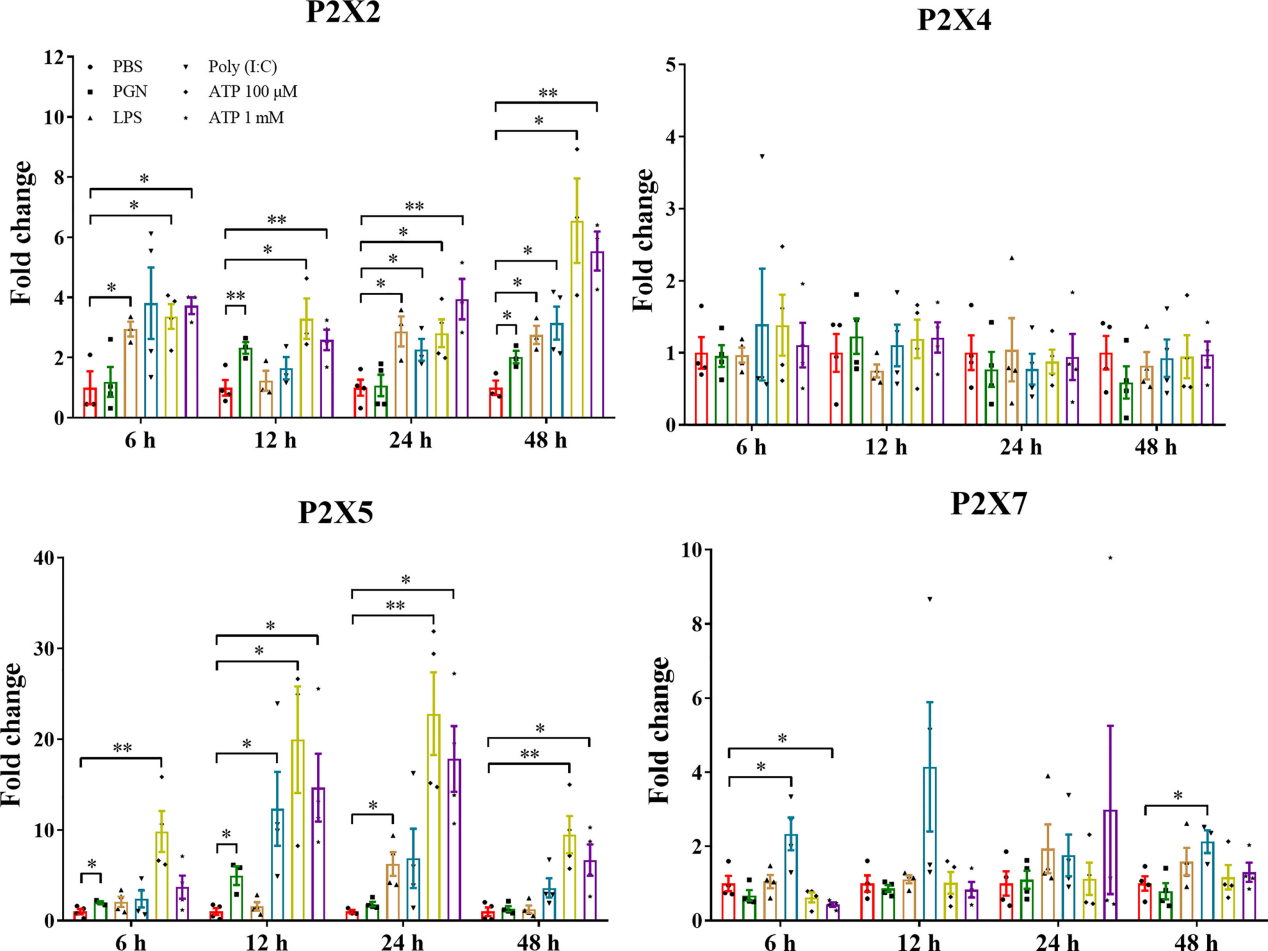
Expressions of *Lm*P2Xs in primary head kidney leucocytes after stimulation with PGN, LPS, poly (I:C) or ATP. Primary head kidney leukocytes were isolated from the spotted sea bass head kidney and stimulated with PGN (50 μg/mL) LPS (100 μg/mL), LPS (100 μg/mL), Poly (I:C) (50 μg/mL), 100 μM ATP, 1 mM ATP, or PBS (control). The total RNA was extracted for qPCR analysis. The relative expression levels of target genes were normalized to that of EF1α and fold change was obtained by comparing the relative gene expression level of the treated group and control group (defined as 1). The data are shown as mean +SEM (N=3). *P<0.05 and **P<0.01 are considered significant difference.

The expressions of *LmP2X*s were investigated in primary head kidney leucocytes in response to PGN, LPS, Poly (I:C), and ATP (**Figure 7**). PGN, LPS, and Poly (I:C) are the major component of bacterial cell walls, bacterial endotoxin, and viral mimetic respectively, which are used to mimic bacterial and viral-mediated immune responses (37, 40). Here, we found that *LmP2X2* and *LmP2X5* were induced after all five kinds of stimulation and were most highly up-regulated by ATP stimulation. For *LmP2X7*, Poly (I:C) stimulation induced its expression at 6 h and 48 h, whereas ATP stimulation inhibited its expression at 6 h. In addition, there were no significant changes in *LmP2X4* expression after all the stimulation.

### 3.6 Subcellular localization of the four P2X receptors

To determine the subcellular localization of *Lm*P2Xs, plasmids pEGFP-N1-*Lm*P2Xs were constructed to express the GFP-tagged *Lm*P2X2, *Lm*P2X4, *Lm*P2X5, and *Lm*P2X7 fusion protein in HEK 293T cells, respectively. As shown in **Supplementary Figure 3**, in HEK 293T cells transfected with expression plasmids, GFP fluorescence were mainly located on the cell membrane, while in HEK 293T cells transfected with empty plasmids, GFP fluorescence was mainly located in the intracellular area.

### 3.7 Effect of activated *Lm*P2Xs on the expressions of inflammation-associated genes

**Figure 8:**
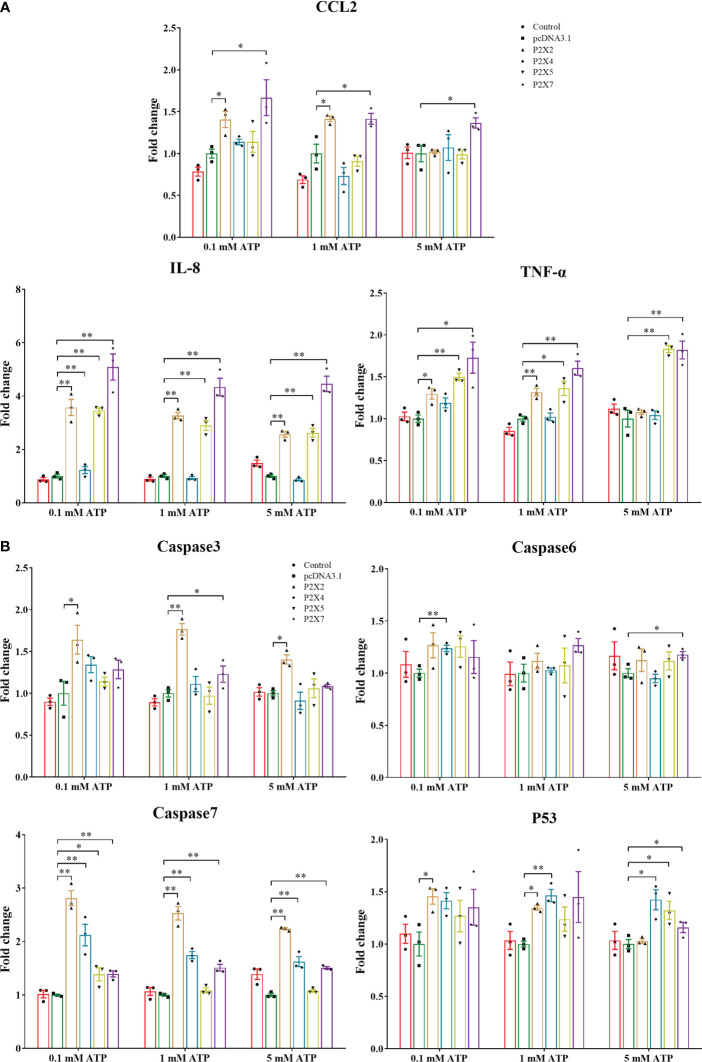
Effect of activated *Lm*P2Xs on expressions of pro-inflammatory cytokines **(A)** and apoptosis-related genes **(B)** in the HEK 293T cells. HEK 293T cells were transfected with pcDNA3.1-*Lm*P2Xs or pcDNA3.1. After 24 h, the cells were stimulated with (0.1 mM, 1 mM, or 5 mM) and were collected after 6 h. The empty plasmid transfected cells by PBS stimulation were served as control. The relative expression levels of target genes were normalized to that of βactin and fold change was obtained by comparing the relative gene expression level of the treated group and control group (defined as 1). The data are shown as mean +SEM (N=3). *P<0.05 and **P<0.01 are considered significant difference.

The mRNA expressions of pro-inflammatory cytokines (*CCL2*, *IL-8*, and *TNF-α*) and apoptosis-related genes (*caspase3*, *caspase6*, *caspase7*, and *P53*) were analyzed in *Lm*P2Xs-overexpressing HEK 293T cells after ATP stimulation (**Figure 8**). Compared to cells transfected with empty plasmids, the mRNA expressions of all 7 genes were significantly upregulated in cells overexpressing *Lm*P2X7, and the mRNA expressions of all genes except caspase6 were also upregulated in cells overexpressing *Lm*P2X2; However, cells overexpressing *Lm*P2X2 were more sensitive to low concentration of ATP, whereas cells overexpressing *Lm*P2X7 were sensitive to high concentration of ATP (**Figure 8**). Meanwhile, overexpression of *Lm*P2X5 also upregulated the mRNA expressions of *IL-8* and *TNF-α* by different concentrations of ATP stimulation, but overexpression of *Lm*P2X4 only up-regulated the *IL-8* mRNA expression following 0.1mM ATP stimulation (**Figure 8A**). In addition, overexpression of *Lm*P2X4 or *Lm*P2X5 up-regulated the mRNA expressions of *caspase7* and *P53* upon ATP stimulation, but had no effect on *caspase3* (**Figure 8B**). Taken together, these results demonstrated that activated *Lm*P2Xs can upregulate the expressions of inflammation-associated genes to various degrees.

### 3.8 Effect of activated *Lm*P2Xs on the NF-κB pathway

**Figure 9:**
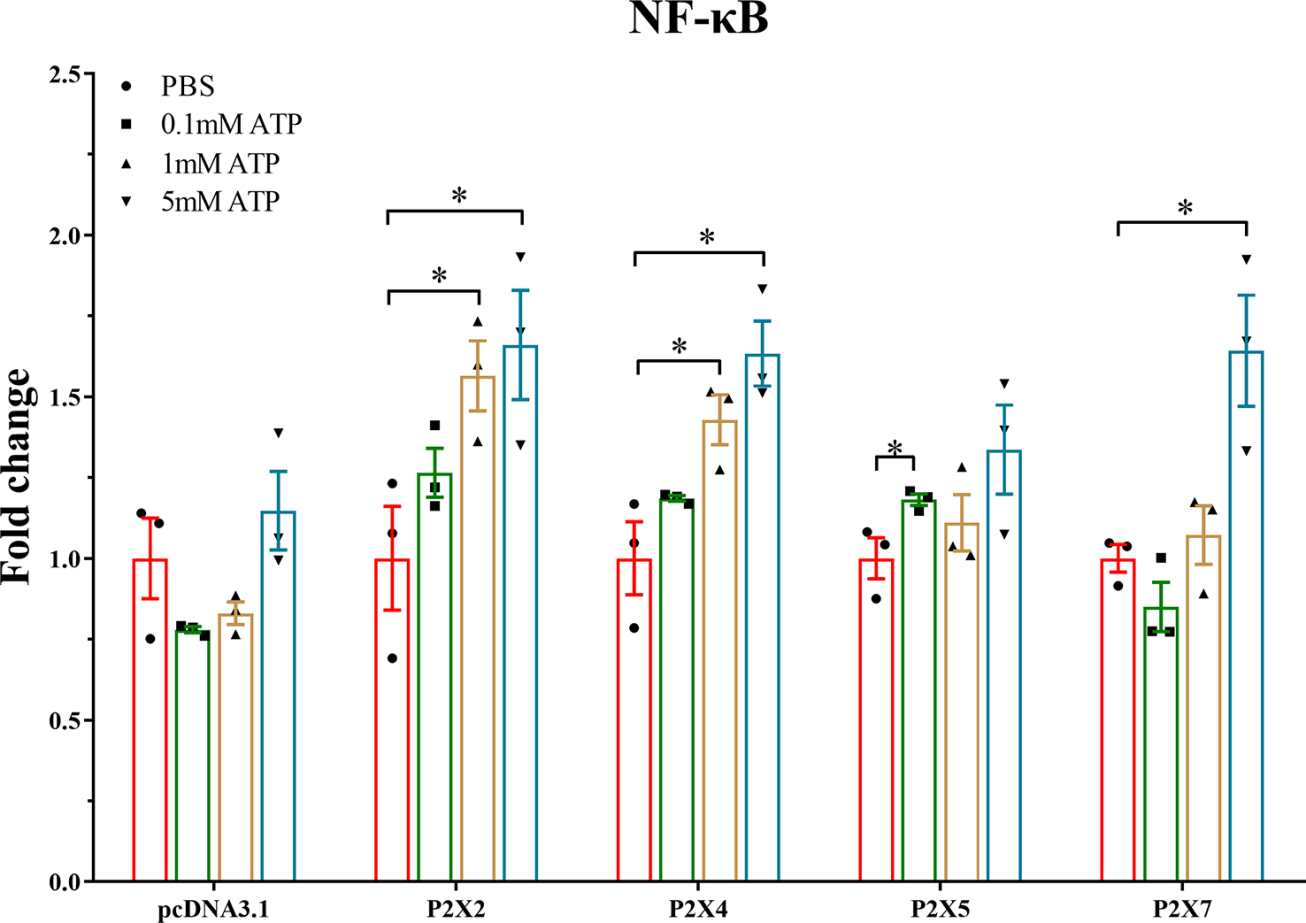
Effect of activated *Lm*P2Xs on activity of NF-κB promoter in HEK 293T cells. The pGL4.32 and pRL-TK (negative control) were co-transfected with pcDNA3.1-*Lm*P2Xs plasmid or pcDNA3.1 plasmid into HEK 293T cells for 24 h. Then the cells were stimulated with ATP (0.1 mM, 1 mM, or 5 mM) or PBS (control) and collected after 6 h. Fold change was obtained by comparing the relative Luciferase activity level of the stimulated group and control group (defined as 1). The data are shown as mean +SEM (N=3). *P<0.05 is considered significant difference.

Luciferase promoter assay was used to investigate the effect of activated *Lm*P2Xs on the NF-κB pathway. As shown in **Figure 9**, in *Lm*P2Xs-expressing cells, ATP stimulation resulted in significant increases of the luciferase activity compared to the control group (PBS). In detail, the NF-κB promoter activity was significantly up-regulated in *Lm*P2X2 or *Lm*P2X4-overexpressing HEK 293T cells after 1 mM and 5 mM ATP stimulation, in *Lm*P2X5-overexpressing HEK 293T cells after 0.1 mM stimulation and in *Lm*P2X7-overexpressing HEK 293T cells after 5 mM ATP stimulation. By contrast, ATP stimulation had no significant influence on the luciferase activity in pcDNA3.1-expressing cells. Taken together, these results suggested that ATP can activate the NF-κB pathway by interacting with *Lm*P2Xs.

## 4 Discussion

As important members of the inflammation and immune response, P2X receptors have been well-studied in mammals (4), however, their immunological functions in fish remain largely unclear. Therefore, their roles in fish inflammation and innate immunity need to be further investigation. In this study, four P2X receptors were characterized in spotted sea bass (*Lateolabrax maculatus*), which were named *LmP2X2*, *LmP2X4*, *LmP2X5*, and *LmP2X7*. They share conserved structures, including, a protein kinase C (PKC) site, ten conserved cysteine residues in the extracellular domain, and a YXXXK motif (**Figure 1**), which are critical for the expression and function of P2X receptors (41–43). Next, we found that they possess conserved genomic organization and gene synteny with their homologues in vertebrates (**Figures 3**, **4**). Structural conservation of the four P2X receptors suggested that they may perform similar functions during evolution. As is known to all, teleost specific whole genome duplication (TSWGD) had resulted in the expansion of gene paralogues (44). Interestingly, although nine genes with high homology for different P2X receptors have been isolated in zebrafish (27, 45), no homologue of *P2X6* has been identified in fish to date. Given the limited research on P2X receptors in fish, the presence or absence of *P2X6* in teleost fish need to be clarified in future studies.

In healthy spotted sea bass, all four *LmP2X*s were constitutively expressed in tissues examined, with lower expressions in head kidney and spleen, even though they are important immune tissues in fish. Similarly, the lower expressions of *P2X2* and *P2X4* were found in head kidney and spleen of Japanese flounder (31, 32). In addition, *P2X7* was also relatively lower expressed in the two tissues of Japanese flounder (30) and gilthead seabream (28). These results suggest a similar expression pattern of P2X receptors in different fish. In addition, their expressions were higher in the mucosal immune tissue intestine or gill, suggesting that they may play an important immune function in defending against pathogenic invasion. On balance, we found that all *LmP2X* were co-expressed in various tissues, but with tissue preferences, demonstrating that multiple P2X receptors may function synergistically or complementarily, but in given tissues, different P2X receptors may contribute differently in the extracellular ATP-mediated immune response. However, these possibilities need to be further examined in the future. On the other hand, studies in mammals have shown that different P2X receptors are involved in regulating a variety of neural and physiological processes (46). In our study, different *LmP2X* were expressed at higher levels in brain, muscle or liver, indicating that they might be involved in physiological processes similar to those in mammals.

Previous studies have demonstrated that the expressions of mammalian P2X receptors can be up-regulated in tissues and immune cells after viral or bacterial infection or PAMP or ATP stimulation (4, 5). In fish, *P2X2* and *P2X4* have also been found significantly up-regulated after various TLR agonists (LPS, Poly (I:C) and zymosan) stimulation and bacterial infection in Japanese flounder (31, 32). In addition, *P2X7* expression was up-regulated after bacterial infection and PAMP stimulation in Japanese flounder and ayu (29, 30). The expressions of *P2X7*-like receptor in the diploblastic coral and the invertebrate chordate amphioxus were also significantly challenged after LPS and ATP stimulation (47). Similarly, in that study, the expressions of all four *LmP2X*s were changed significantly by stimulation with LPS, Poly (I:C) or *E. tarda* infection *in vivo*, with varying degrees in different tissues (**Figure 6**). Furthermore, *LmP2X2* and *LmP2X5* were also induced by stimulation with PAMP and ATP in primary head kidney leukocytes (**Figure 7**). Poly (I:C) is a synthetic double stranded RNA analog of viral nucleotide, and LPS as well as PGN are the main component of the outer membrane of bacteria. *E. tarda* is an intracellular bacterial pathogen and has been reported to infect vertebrates from fish to mammals (48). The severe disease and fatal cases by *E. tarda* have been reported in a wide range of fish species (37, 49). Thus, our results indicated that all four *LmP2X*s were probably involved in both antiviral and antibacterial immune responses but with tissue specificity. Noteworthy, to our knowledge, this is the first report on the involvement of P2X5 in fish immunity.

In mammalian, numerous studies indicated that extracellular ATP promotes the release of pro-inflammatory cytokines and chemokines, induces apoptosis, and activates MAPK and NF-κB through the activation of different P2X receptors (4–7). Similarly, extracellular ATP has been shown to induce the mRNA expressions of pro-inflammatory cytokines and immune-related genes, and to exert a pro-apoptotic effect by promoting the expressions and enzymatic activities of several caspases in fish immune cells (33, 50–53). Activated P2X7 induced the mRNA expressions of *IL-1β* and *IL-6* in Japanese flounder head kidney primary cells (30) and mediated cell death of ayu macrophages (29). The binding of coral and amphioxus P2X7L and flounder P2X7 to extracellular ATP has been demonstrated to induce the both mRNA and protein expressions of IL-1β, IL-6, IL-8, and CCL2 in HEK 293T cells (47). In addition, some fish genes have been shown to modulate apoptosis in HEK 293T cells by regulating the mRNA expressions of apoptosis-related genes (39, 54). Here, we first demonstrated that *Lm*P2X2, *Lm*P2X4, *Lm*P2X5 and *Lm*P2X7 were localized on the cellular membrane and can be expressed in HEK 293T cells (**Supplementary Figure 3**). Then, we found that *Lm*P2Xs activated by extracellular ATP differentially up-regulated the mRNA expressions of pro-inflammatory cytokines (*CCL2*, *IL-8*, and *TNF-α*) and apoptosis-related genes (*caspase3*, *caspase6*, *caspase7*, and *P53*) in HEK 293T cells (**Figure 8**), suggesting that activated *Lm*P2Xs may be involved in inflammation and innate immune response by regulating the expressions of inflammation-associated genes. However, the direct immunological roles of P2X receptors, especially those other than P2X7, in fish cells needs to be further investigated.

We further investigated the effect of activated *Lm*P2Xs on the NF-κB promoter, which is a critical signaling pathway controlling the expressions of inflammation-related genes (55). The results showed that ATP-activated *Lm*P2Xs significantly increased the NF-κB promoter activity (**Figure 9**), implicating that ATP could activate the NF-κB pathway by interacting with *Lm*P2Xs. Previous study also showed that extracellular ATP can induce NF-κB p65 subunit (*p65*) expression in Japanese flounder immune cells (53). As activation of the NF-κB pathway induces expressions of pro-inflammatory cytokines, these results provide strong evidence for activated P2X receptors up-regulate the expressions of inflammation-related genes *via* the NF-κB pathway in fish. However, their functional mechanisms need to be further clarified in the future.

P2X receptors have been demonstrated to have different affinities for extracellular ATP in mammals (7). All P2X receptors have high ATP-affinities (EC_50 = _0.05-30 μM) except for P2X7, which has a low affinity for ATP (EC_50_ > 100 μM) (7, 56). However, in zebrafish, it was shown that P2X2 did not respond to low levels of ATP (30 μM) (27) and that P2X4 had a significantly lower affinity for ATP (EC_50 = _274 ± 48 μM) than mammals (26). Japanese flounder P2X7 also exhibited a much lower ATP-affinity (EC_50 = _790 ± 81 μM) than the P2X7 of mammalians (30). In addition, mouse P2X4 promoted ATP-dependent pore formation only at low ATP concentrations (≤ 1 mM) (57), while zebrafish P2X4 could be activated by higher ATP concentrations (≥ 3 mM) (26). Interestingly, in our study, *LmP2X2* and *LmP2X5* were induced by higher concentrations of ATP in head kidney primary leukocytes (**Figure 7**). At the same time, although *Lm*P2X2, *Lm*P2X4 and *Lm*P2X5 were more sensitive to ATP than *Lm*P2X7, all four *Lm*P2Xs were activated by higher concentrations of ATP (≥ 1 mM) to perform their functions (**Figures 8**, **9**). Taken together, the ATP-affinity of fish P2X receptors may be much lower than that of mammals. On the other hand, a number of key sites and structures of different mammalian P2X receptors to perform their functions have been reported (46). For example, it has been demonstrated that the TM2 structural domain of P2X7 has a key role in receptor function and the residues important for full P2X7 function have also been identified in mouse (57). However, these issues have not been well studied in fish. Therefore, the concentration range of ATP sensitivity and the key sites for the function of different fish P2X receptors need to be determined in the future to better explain these issues.

In summary, four P2X receptors (*P2X2*, *P2X4*, *P2X5*, and *P2X7*) were identified in spotted sea bass. They were constitutively expressed in the tissues and significantly upregulated by E. *tarda* infection and PAMP and ATP stimulation. Furthermore, activated *Lm*P2Xs induced the expressions of inflammation-related genes, and significantly increased the NF-κB promoter activity. Thus, these four P2X receptors participated in innate immune response of spotted sea bass probably by regulating the expressions of inflammation-related genes. These results provide new evidence for the involvement of P2X receptors in fish immunity, which will be helpful in further understanding the innate immune response mediated by extracellular ATP in fish.

## Data availability statement

The data presented in the study are deposited in the NCBI repository, accession numbers OP651016, OP651019, OP651020, and OP651021.

## Ethics statement

The animal study was reviewed and approved by the ethics committee of laboratory animals of Shanghai Ocean University (SHOU-DW-2019-012).

## Author contributions

ZS: investigation, methodology, data curation, and writing the original draft. QG: conceptualization, funding acquisition, project administration, supervision, and reviewing and editing. YW: supervision and reviewing and editing. ZZ: reviewing and editing. YC, CX, JG, and DL: investigation and methodology. All authors contributed to the article and approved the submitted version.
