# Supplementary material for: Activated P2X receptors can up-regulate the expressions of inflammation-related genes via NF-κB pathway in spotted sea bass (Lateolabrax maculatus)
Source: Front Immunol. 2023 May 4;14:1181067. doi: 10.3389/fimmu.2023.1181067 (PMC10193947; doi:10.3389/fimmu.2023.1181067)
Supplement: Supplementary file 1 [file DataSheet_1.docx]

Supplementary Material

Activated P2X Receptors can Up-regulate the Expressions of Inflammation-Related Genes via NF-κB Pathway in Spotted Sea Bass (*Lateolabrax maculatus*).

**Zhaosheng Sun, Qian Gao^*^, Youchuan Wei^*^, Zhigang Zhou, Yuxi Chen, Chong Xu, Jiaqi Gao and Danjie Liu**

*** Correspondence:** Qian Gao: [qgao@shou.edu.cn](mailto:qgao@shou.edu.cn) and Youchuan Wei: weiyc@gxu.edu.cn

**Supplementary Table 1. Primers used for cloning and real-time PCR.**

| Primers | Sequence (5´ to 3´) | Application |
| --- | --- | --- |
| *Lm*P2X2-F1 | GAAGCGTTCAGTTTCTCGTGAT | Sequence validation |
| *Lm*P2X2-R1 | CAGGTCGGAGTGGTTTGAGCC | Sequence validation |
| *Lm*P2X2-3F1 | TACATATTCCTTCCGTCGTCTTGA | 3´-RACE |
| *Lm*P2X2-3F2 | GACCATCATCAGCACCGTGAC | 3´-RACE |
| *Lm*P2X2-5R1 | TACTCAGCGCAGGTCCCTTG | 5´-RACE |
| *Lm*P2X2-5R2 | TTATCACATCACCGCCCTCTG | 5´-RACE |
| *Lm*P2X2-F2 | ACTGGAGCCAAGCAGGAGCA | Verify the CDS |
| *Lm*P2X2-R2 | TTACAGCGGTACACCTTCTGAC | Verify the CDS |
| *Lm*P2X4-F1 | CTTCTGGTGGTGCTGTATGTGGT | Sequence validation |
| *Lm*P2X4-R1 | TCCATCTGCGGTCTTGTAGTATTT | Sequence validation |
| *Lm*P2X4-3F1 | GCAATGGCATACAGACGGGACT | 3´-RACE |
| *Lm*P2X4-3F2 | ATAGACACTAAGCTGCCCAAACG | 3´-RACE |
| *Lm*P2X4-5R1 | CGTTTGGGCAGCTTAGTGTCTAT | 5´-RACE |
| *Lm*P2X4-5R2 | CCGTCTGTATGCCATTGCCTC | 5´-RACE |
| *Lm*P2X4-F2 | GATTCGAGCTCGGTACCCGG | Verify the CDS |
| *Lm*P2X4-R2 | TGTTAACAATTTCGGAGCAGGTC | Verify the CDS |
| *Lm*P2X5-F1 | CTGAGTCTGGTCTGCTCGTGTG | Sequence validation |
| *Lm*P2X5-R1 | ACAAAGCCAGTCCTGAAGCA | Sequence validation |
| *Lm*P2X5-3F1 | TTGGTGGCTCCATTGGCATTA | 3´-RACE |
| *Lm*P2X5-3F2 | ACATCGGCATCTCCAACAAG | 3´-RACE |
| *Lm*P2X5-5R1 | CCAGGCGTAGATTTCACAAGTA | 5´-RACE |
| *Lm*P2X5-5R2 | CAGCCACTACCATCATTCCTTCTT | 5´-RACE |
| *Lm*P2X5-F2 | TAGCCTGTATTTTGATGGTGGGA | Verify the CDS |
| *Lm*P2X5-R2 | GCGAAGAACCAGGTCAGTGATA | Verify the CDS |
| *Lm*P2X7-F1 | ACGGAGCCATCCTGCTGTTTATC | Sequence validation |
| *Lm*P2X7-R1 | TCAGCAGGTGGAGACAGAGGGT | Sequence validation |
| *Lm*P2X7-3F1 | TCGGACAGGCAGGAAGGTT | 3´-RACE |
| *Lm*P2X7-3F2 | TGTTCAGCCACGCAAGGAG | 3´-RACE |
| *Lm*P2X7-5R1 | CCTCAGGTAGGCGTCGTTCAT | 5´-RACE |
| *Lm*P2X7-5R2 | GACCGGACACCACGCAGAAA | 5´-RACE |
| *Lm*P2X7-F2 | GAGACCTCCTGAAACCTGAACCC | Verify the CDS |
| *Lm*P2X7-R2 | CCCTGCTCTATAATGGGGAAACTC | Verify the CDS |
| 3´CDS | AAGCAGTGGTATCAACGCAGAGTAC(T)_30_VN | 3´-RACE |
| UPM-long | CTAATACGACTCACTATAGGGCAAGCAGTGGTATCAACGCAGAGT | 3´-RACE |
| UPM-short | CTAATACGACTCACTATAGGGC | 3´-RACE |
| NUP | AAGCAGTGGTATCAACGCAGAGT | 3´-RACE |
| APG | CCAGACTCGTGGCTGATGCA(G)16 | 5´-RACE |
| AP | CCAGACTCGTGGCTGATGCA | 5´-RACE |
| *Lm*P2X2-qF | GAGCTCTGCAGGACTGGAGGA | Real-time PCR |
| *Lm*P2X2-qR | GGGCTCCTTGATCACCTCATC | Real-time PCR |
| *Lm*P2X4-qF | CGTCATTGTATTTGGAACTGCAGG | Real-time PCR |
| *Lm*P2X4-qR | TGGTGCCCATTGTCATTGATTC | Real-time PCR |
| *Lm*P2X5-qF | AACGGGGTTTAACTTCAGACACAC | Real-time PCR |
| *Lm*P2X5-qR | TGTCACAGAAGAAAGCTCCAGCAC | Real-time PCR |
| *Lm*P2X7-qF | CGAGACAAACAGAAGTGCATCGTC | Real-time PCR |
| *Lm*P2X7-qR | TTCGGGTCGGGTTTGTGGT | Real-time PCR |
| *Lm*EF1α-qF | ATCTCTGGATGGCACGGAGA | Real-time PCR |
| *Lm*EF1α-qR | CAGTGTGGTTCCGCTAGCAT | Real-time PCR |
| *Hs*CCL2-qF | AGAATCACCAGCAGCAAGTGTCCC | Real-time PCR |
| *Hs*CCL2-qR | TTCTTGGGTTGTGGAGTGAGTGTTC | Real-time PCR |
| *Hs*IL8-qF | GCTCTCTTGGCAGCCTTCC | Real-time PCR |
| *Hs*IL8-qR | GGGTGGAAAGGTTTGGAGTATGTCT | Real-time PCR |
| *Hs*TNFα-qF | CCCCAGGGACCTCTCTCTAA | Real-time PCR |
| *Hs*TNFα-qR | CTCAGCTTGAGGGTTTGCTAC | Real-time PCR |
| *Hs*casp3-qF | CCTGGTTCATCCAGTCGCTT | Real-time PCR |
| *Hs*casp3-qR | TCTGTTGCCACCTTTCGGTT | Real-time PCR |
| *Hs*casp6-qF | CACCAACATAACTGAGGTGGATG | Real-time PCR |
| *Hs*casp6-qR | AGGAGGAGCCATATTTTCCCA | Real-time PCR |
| *Hs*casp7-qF | GACCGAGCTTGATGATGGC | Real-time PCR |
| *Hs*casp7-qR | CTGGAACCGTGGAATAGGC | Real-time PCR |
| *Hs*P53-qF | GCGCACAGAGGAAGAGAATC | Real-time PCR |
| *Hs*P53-qR | CTCGGAACATCTCGAAGC | Real-time PCR |
| *Hs*βactin-qF | ATCGTGCGTGACATTAAGGAGAAG | Real-time PCR |
| *Hs*βactin-qR | AGGAAGGAAGGCTGGAAGAGTG | Real-time PCR |

**A**

actggagccaagcaggagcagagtgagaaagaaaaatagtgagtgaaataaaaaggtagaag 62
cagagagatagaatcatagagaaattagacttcaccaggacatttcaagat**ATG**TGTGAA 122
                                                    M  C  E  3
TTGTTTTCCAAGTTCATCATGGGGCTCCGTGAATTTATTAAGGAGTACTATCTTGGTTTC 182
 L  F  S  K  F  I  M  G  L  R  E  F  I  K  E  Y  Y  L  G  F  23
TGGGACTATGAGACACCAAAGGTGATGGTAGTTAAAAATAAAACTCTTGGAGTCATATAC 242
 W  D  Y  E  T  P  K  V  M  V  V  K  N  K  T  L  G  V  I  Y  43
AGAAGCGTTCAGTTTCTCGTGATCACCTATTTCATCTGGTATGTCTTCATAAGTCAGAAA 302
 R  S  V  Q  F  L  V  I  T  Y  F  I  W  Y  V  F  I  S  Q  K  63
GCTTACCAAGAGAGTGAAACTCGTCCAGAAAGCTCAGTTTACACCCTCATGAAAGGCACA 362
 A  Y  Q  E  S  E  T  R  P  E  S  S  V  Y  T  L  M  K  G  T  83
GCAGTTCATGGAGATGATATCTTGGACACTGTGGAGTACGCTCGACCCTCAGAGGGCGGT 422
 A  V  H  G  D  D  I  L  D  T  V  E  Y  A  R  P  S  E  G  G  103
GATGTGATAAGCACAATATTGAGACGAGAAGTAACGTATGATCAGATGCAAGGGACCTGC 482
 D  V  I  S  T  I  L  R  R  E  V  T  Y  D  Q  M  Q  G  T  C  123
GCTGAGTATTTCAATGTTGCCAATGCCAACTGTACAACAGACTCTGACTGTGTCCAGGGA 542
 A  E  Y  F  N  V  A  N  A  N  C  T  T  D  S  D  C  V  Q  G  143
GAGGCTGACTTTGATGGCCATGGCAGAAGGACCGGAAGATGTGTTCAGTACTACAACCAC 602
 E  A  D  F  D  G  H  G  R  R  T  G  R  C  V  Q  Y  Y  N  H  163
ACCTTCAAAACCTGTGAGATCCAAACCTGGTGTCCTATTGAGGAGTACGCTGTAGTACGA 662
 T  F  K  T  C  E  I  Q  T  W  C  P  I  E  E  Y  A  V  V  R  183
GAACCAGCATTAGTGGAGGCCATCAATTTCACAGTGTTCATCAGGAACTCCATCCACTTC 722
 E  P  A  L  V  E  A  I  N  F  T  V  F  I  R  N  S  I  H  F  203
CCCAAATTTAAAGTGCTGAGGGGAAATATCAAAGATGCTTCAACCAAGCATGACATGCAG 782
 P  K  F  K  V  L  R  G  N  I  K  D  A  S  T  K  H  D  M  Q  223
AAATACCTCAGGAAGTGTCATTATAATGAGGAGAAAGATCCCTACTGTCCAAACTTCCGC 842
 K  Y  L  R  K  C  H  Y  N  E  E  K  D  P  Y  C  P  N  F  R  243
CTGGGCTACATCGCAGATCAAGCGAGGGAGAATTTCAATGAGCTCTGCAGGACTGGAGGA 902
 L  G  Y  I  A  D  Q  A  R  E  N  F  N  E  L  C  R  T  G  G  263
GTGATAGGAGTTTTCATCAACTGGAAGTGTAACCTGGACCTGGATCCTTCACACTGTAAA 962
 V  I  G  V  F  I  N  W  K  C  N  L  D  L  D  P  S  H  C  K  283
CCTACATATTCCTTCCGTCGTCTTGATCTGCGAAAGGACCAGGCCAACTCTGGTTACTAT 1022
 P  T  Y  S  F  R  R  L  D  L  R  K  D  Q  A  N  S  G  Y  Y  303
TACAGGTTTGCCAAATATTACAGCAAGAATGGAGAAGAGTCTCGGACACTTATCAAAGCC 1082
 Y  R  F  A  K  Y  Y  S  K  N  G  E  E  S  R  T  L  I  K  A  323
TATGGCATCCGTCTGGATGTCATAGTTCACGGACATGCTGGTAAATTCAGTCCCATCCCG 1142
 Y  G  I  R  L  D  V  I  V  H  G  H  A  G  K  F  S  P  I  P  343
ACCATCATCAGCACCGTGACTGCTATGACTTCAGTCGGGATTTGTACCATTATCTGTGAC 1202
 T  I  I  S  T  V  T  A  M  T  S  V  G  I  C  T  I  I  C  D  363
TGGATCATGCTGACTTTTATTGACAAGAATGAAATCTACAGCGAGAGAAAGTTTGATGAG 1262
 W  I  M  L  T  F  I  D  K  N  E  I  Y  S  E  R  K  F  D  E  383
GTGATCAAGGAGCCCGCGGTGCCAGTTTCCACGGAGCTCAACTACATGTACAGCTACGGC 1322
 V  I  K  E  P  A  V  P  V  S  T  E  L  N  Y  M  Y  S  Y  G  403
TCAAACCACTCCGACCTGTCAGAAGGTGTACCGCTG**TAA**tgtcgtcacagctgcctccgt 1382
 S  N  H  S  D  L  S  E  G  V  P  L  -  415
cctcactggcctagagtgtggactctcctccatcagctgactgtggcaactctgagtggt 1442
ttctcaggtgtctgcaactgctcggttatggactgccagcggttgctatgctgttttgca 1502
gtgggaataaactggcagagaaccatagtgacagagggcctcttttgaaagacagccatt 1562
tctctcaaagtctctgcctgtgcagtggattcagttcatagatcatctcataccccgatg 1622
gaccccaactctacaacaatgcaattaattctttctctttcacttgacatgacttcagaa 1682
gacggcagccccaattacaaaatgtgtttattttctccctataaatagaaactaaaccat 1742
ttcatagcagaaaaatatttgagtacatagtaaggagaaactatgcctatttctatcaat 1802
gtattgcacatattcaatgc**aataaa**tgtcctctagcaactcaagattcgaaaaaaaaaa 1862
aaaaaaaaaaaaaaaaaa 1880

**B**

catcactggtgtgtgtggtgcagcg**ATG**AGCAGGACTGCAGGCTGCTGCCAGCGCTGTCTG 61
                          M  S  R  T  A  G  C  C  Q  R  C  L  12

CATTTTGTGTTTGATTATGAAACACCAAAAACACTGGTTATTCCAAACTTAAGGGTGGGA 121

 H  F  V  F  D  Y  E  T  P  K  T  L  V  I  P  N  L  R  V  G  32
TGCGTCTTCAGGTTCACCCAGCTTCTGGTGGTGCTGTATGTGGTGGGGTATGTGTGTGTG 181

 C  V  F  R  F  T  Q  L  L  V  V  L  Y  V  V  G  Y  V  C  V  52
GTGCAGAAGGCCTACCAGGTTACAGACTCTGTCCTCAGTACCGTCACCACCAAAGTGAAA 241

 V  Q  K  A  Y  Q  V  T  D  S  V  L  S  T  V  T  T  K  V  K  72
GGTTTTGCCTTCACCAACACGTCTGACTTGGACCCCACGTTTTGGGACGTGGCTGATTAT 301

 G  F  A  F  T  N  T  S  D  L  D  P  T  F  W  D  V  A  D  Y  92
GTTATCCCACCTCAGGGTGATAATTCATTCTTTGTGTTGACGAATATGGTTGTTACCCCC 361

 V  I  P  P  Q  G  D  N  S  F  F  V  L  T  N  M  V  V  T  P  112
AATCAAACACAGTCACGTTGTCCTGAGCTGCCAAACCCGTCAACTACTTGTGTGGATGAC 421

 P  E  L  P  N  P  S  T  T  C  V  D  D  C  N  Q  T  Q  S  R  132
TGTGACTGTATCGAGGGCTACAGTAATCCTCGAGGCAATGGCATACAGACGGGACTGTGT 481

 C  D  C  I  E  G  Y  S  N  P  R  G  N  G  I  Q  T  G  L  C  152
GAGAACTACTCCACGACTGTCCGGACCTGTCAAGTGCTCTCATGGTGCCCTCTTGAAATA 541

 R  T  C  Q  V  L  S  W  C  P  L  E  I  D  E  N  Y  S  T  T  172
GACACTAAGCTGCCCAAACGTGCACCGCTGGCTGCAGCAGAGAACTTCACCGTGTTGATC 601

 V  T  K  L  P  K  R  A  P  L  A  A  A  E  N  F  T  V  L  I  192
AAAAACAGCATCACATACCCGAATTTCAACGTTCACAGAAGAAACATCCTACCACACATT 661

 K  N  S  I  T  Y  P  N  F  N  V  H  R  R  N  I  L  P  H  I  212
AACTCCTCATACCTGAAGAGGTGTGAATTTAATCGTACAACGGACCCCGACTGCCCCATA 721

 N  S  S  Y  L  K  R  C  E  F  N  R  T  T  D  P  D  C  P  I  232
TTCCGCCTCAAACACATCGTTTCAGAGGCTGGGGAAGAATTTCAAGACATAGCTGTGAAG 781

 S  E  A  G  E  E  F  Q  D  I  A  V  K  F  R  L  K  H  I  V  252
GGCGGTGTCCTCGGTATTATTATTGACTGGAGCTGTGACCTGGACTGGTGGGCAGGGAAG 841

 G  G  V  L  G  I  I  I  D  W  S  C  D  L  D  W  W  A  G  K  272
TGTTACCCCAAGTACAGCTTCCGCAGGCTGGACAACAAATATCCTGTCAATAATGTGGCC 901

 R  R  L  D  N  K  Y  P  V  N  N  V  A  C  Y  P  K  Y  S  F  292
CCAGGATACAACTTCAGGTTTGCAAAATACTACAAGACCGCAGATGGAGAGGAAACTAGA 961

 P  G  Y  N  F  R  F  A  K  Y  Y  K  T  A  D  G  E  E  T  R  312
ACCTTGATCAAAGCATACGGGATCCGGTTTGACGTCATTGTATTTGGAACTGCAGGGAAG 1021

 T  L  I  K  A  Y  G  I  R  F  D  V  I  V  F  G  T  A  G  K  332
TTTGGAATTATTCCGACCATTGTCAACTTGGGTGCAGCATTATCGTTCCTCAGTTTGGTT 1081

 F  G  I  I  P  T  I  V  N  L  G  A  A  L  S  F  L  S  L  V  352
CCAGTGGTTTCTGACTGGTTTATGTTGACATGCATGAAGAAAAGAGATTTTTACAGCAAA 1141

 P  V  V  S  D  W  F  M  L  T  C  M  K  K  R  D  F  Y  S  K  372
CATAAAGTCACATATCTGAGTGAGGACACTGAGTCTGAATCAATGACAATGGGCACCAGC 1201

 H  K  V  T  Y  L  S  E  D  T  E  S  E  S  M  T  M  G  T  S  392
TACGGGACCCAG**TAG**catggtcgcatcgaaatgggaacatttttctgcagaggacctgct 1261

 Y  G  T  Q  -  396
ccgaaattgttaacatgaaaacaaatatattcaatatgaattcttc**aataaa**actggttg 1321
aatgtgtaaaaaaaaaaaaaaaaaaaaaaaa 1352

**C**

tgcactcccatcgggccaacccgtccaaaaatgaacacatgcctactttaagtagcctgta 61
ttttgatggtgggataagcgtctttaaagcaccgtgtcattaaggcaccgggccgtctgc 121
ggtgagcgtcggtactgtaactgtccgcagcggactgcgtgtgcgccgccaccgccacac 181
tccgcacacaccgggtccgcaggcggcggcagg**ATG**GCCGGGAGCTTCTGCAGAGGACGC 241
                                  M  A  G  S  F  C  R  G  R  9
TTCCTCTCCTTGTTCGACTACAAAACGGAAAAATACATTGTCGCCAAAAATAAGAAAGTT 301
 F  L  S  L  F  D  Y  K  T  E  K  Y  I  V  A  K  N  K  K  V  29
GGGGTTTTGTACAGACTTATTCAGTTATCTATCTTCGGTTACATTATAGGGTGGGTTTTC 361
 G  V  L  Y  R  L  I  Q  L  S  I  F  G  Y  I  I  G  W  V  F  49
TTAAGCAAGAAAGGCTACCAGGAGACAGACGAGGCCATCCAGAGTTCTGTCATTACTAAA 421
 L  S  K  K  G  Y  Q  E  T  D  E  A  I  Q  S  S  V  I  T  K  69
CTGAAAGGAGTCTCGGTGACTAACACCACTGAGTCTGGTCTGCTCGTGTGGGGACCGGAG 481
 L  K  G  V  S  V  T  N  T  T  E  S  G  L  L  V  W  G  P  E  89
GACTACGTTATCCCACCACAGGGTGAAGCTGTTCTCTTTGTTGTAACCAATTTCTTAGAG 541
 D  Y  V  I  P  P  Q  G  E  A  V  L  F  V  V  T  N  F  L  E  109
ACTCCAAACCAGAAGCTGGGATACTGTGCTGAGAGCCCCAAAGTGCTGGGCGGCCACTGT 601
 T  P  N  Q  K  L  G  Y  C  A  E  S  P  K  V  L  G  G  H  C  129
CAAGATGACGAGGACTGCAAAGAAGGAATGATGGTAGTGGCTGGTAACGGAATTATGAAT 661
 Q  D  D  E  D  C  K  E  G  M  M  V  V  A  G  N  G  I  M  N  149
GGCCGATGCTTAAGAAAAGATGAAAACTCCACTGGTACTTGTGAAATCTACGCCTGGTGT 721
 G  R  C  L  R  K  D  E  N  S  T  G  T  C  E  I  Y  A  W  C  169
CCCATTGAAAGAAAATTCAAACCACAAGAGCCTCTGCTGACAAACGCTGAAAATTTTACC 781
 P  I  E  R  K  F  K  P  Q  E  P  L  L  T  N  A  E  N  F  T  189
ATCTACATCAAGAATTTCATCCAATTTCCTAAATTCATATTTTCAAAGTACAACGTCCTT 841
 I  Y  I  K  N  F  I  Q  F  P  K  F  I  F  S  K  Y  N  V  L  209
GAAACAACTGATGACTCCTATTTGAGGAAATGCCGGTATGATGAGGAGCTTCACCCCTAC 901
 E  T  T  D  D  S  Y  L  R  K  C  R  Y  D  E  E  L  H  P  Y  229
TGCCCCATCTTTCGCCTGGGAGACATCACCAGGCGAGCTGGATACAACTTCCAGGACATG 961
 C  P  I  F  R  L  G  D  I  T  R  R  A  G  Y  N  F  Q  D  M  249
GCCACATTTGGTGGCTCCATTGGCATTATGATAGAGTGGGACTGTGACCTCGACAAAGGC 1021
 A  T  F  G  G  S  I  G  I  M  I  E  W  D  C  D  L  D  K  G  269
TACTCCAACTGCCATCCACAGTACCACTTTACTCGGCTGGACATCGGCATCTCCAACAAG 1081
 Y  S  N  C  H  P  Q  Y  H  F  T  R  L  D  I  G  I  S  N  K  289
ACCATCGCAACGGGGTTTAACTTCAGACACACTCGGTATTTCAAAAATGCAGCTGGTGAG 1141
 T  I  A  T  G  F  N  F  R  H  T  R  Y  F  K  N  A  A  G  E  309
AGCTATCGATCTCTATTCAAAGTCTACGGTGTCCGTTTTAACATCATGGTGCACGGGAAG 1201
 S  Y  R  S  L  F  K  V  Y  G  V  R  F  N  I  M  V  H  G  K  329
GCCGGGAAGTTCAGCATCATCCCAACTGCCATCAATGTTGCTTCAGGACTGGCTTTGTTG 1261
 A  G  K  F  S  I  I  P  T  A  I  N  V  A  S  G  L  A  L  L  349
GGTGCTGGAGCTTTCTTCTGTGACATGGTCCTTCTCTACCTAATGAAAAAAGGCACTTCT 1321
 G  A  G  A  F  F  C  D  M  V  L  L  Y  L  M  K  K  G  T  S  369
TATCGGGAGAGGAAATTTGAAGGATGCAGCAATAATAAACCATCATCTGAAGACAACAGT 1381
 Y  R  E  R  K  F  E  G  C  S  N  N  K  P  S  S  E  D  N  S  389
GTGGAAGACAGAAAGGAGCAGGAGAATCTCACATCT**TAA**tcttttctctgcatgaaacgg 1441
 V  E  D  R  K  E  Q  E  N  L  T  S  -  401
gtttgtaacatgcccttgttggttatatataaccataaataaaatatatagcagtacagt 1501
ataacatatgctgtactgttgtagaagcttcaccttaaaaataattatttggttgaattt 1561
tacagttttaagatgccaccatattttgccttatttttataatttctatataatataatg 1621
aatagatgttccattaactttataccaggtttagtcataaattgagtgtatttcagcatc 1681
attgtcttgaactgatactgctgcatgcagaccaaatttagtttttctggctgttccttt 1721
ttcactttaactgtttcacgacttgatttggtgtctggtcagacttaaaaaacctgaatt 1801
caaatctcatctttaaaataaactaattcaaatgagtatatatatatacacatatttttt 1861
atttatttatttttctctcatcatgacatactgtgtaaaggttgtaattttgattgctga 1921
ttggataaaaggaaatttttgtaagtcacatttggtgcctcaccttaaaggtgaaacagg 1981
agtggtgactctgacatactgactattacaatgtttatttagaagaaggctgtacagtaa 2041
tgctgtatatatttaatttaaacatcatcatttagaccaaaactcttcacagcggcctca 2101
cacagctcagtgtttatgccacatatcactgacctggttcttcgcctgtgtgtgtatatg 2161
taacctaattaaatgtgca**aataaa**gattttgataggaaaaaaaaaaaaaaaaaaaaaaa 2221
aaaaaaa 2228

**D**

cttcctccgctgcaggaaaccacacagagacctcctgaaacctgaacccagcgagccgcacc 62
aggaccgggaccgggaccaggaccgggaccaagaccgggaccaggaccaggaccgggacc 122
aggaccgggaccatc**ATG**CCGTGCCGCCGTCTTCTCGGTCTGTGTCAGTATGAGACCAAC 182
                M  P  C  R  R  L  L  G  L  C  Q  Y  E  T  N  15
AAGCTGGTCCGGATCCAGAGCGTCCGGCTCGGCTCTCTGAAGTGGAGTCTGAACGGAGCC 242
 K  L  V  R  I  Q  S  V  R  L  G  S  L  K  W  S  L  N  G  A  35
ATCCTGCTGTTTATCTGCATCATGATGCTCTGGAACAGGAAGTACCAGGAGTTTGACCTG 302
 I  L  L  F  I  C  I  M  M  L  W  N  R  K  Y  Q  E  F  D  L  55
GTGGTGAGCTCTGTCACCACCAAGGTGAAGGGTGTCGCCCAGGCCCACCTGCCCGGGGTC 362
 V  V  S  S  V  T  T  K  V  K  G  V  A  Q  A  H  L  P  G  V  75
GGGGACGTGGTCTGGGACGTGGTGGACTACAGCGGGCCCTCGCAGGACAAAAACTCCTTC 422
 G  D  V  V  W  D  V  V  D  Y  S  G  P  S  Q  D  K  N  S  F  95
TTTGTGGTGACCAACGTCATCGTGACAAAGAATCAGAGGCAGGGGAAATGTCCAGAGGTT 482
 F  V  V  T  N  V  I  V  T  K  N  Q  R  Q  G  K  C  P  E  V  115
CCTCTTAAAGGCAGATTGTGTCGCACTGATAAGGACTGTGAGAAGGGCTCCTGGGACCAG 542
 P  L  K  G  R  L  C  R  T  D  K  D  C  E  K  G  S  W  D  Q  135
CAGAGCCACGGGATCCAGACGGGATCGTGTCTGAGGTTTGACGTGTTGAGGAAAACCTGC 602
 Q  S  H  G  I  Q  T  G  S  C  L  R  F  D  V  L  R  K  T  C  155
GAGGTTTCTGCGTGGTGTCCGGTCGAAGCCAGGACGGACCCCCCGAGACCTGCTCTGCTG 662
 E  V  S  A  W  C  P  V  E  A  R  T  D  P  P  R  P  A  L  L  175
GCAGCGGCGGAGAACTTCACAGTCCTCATCAAAAACAACATCAGGTTTCCTGCGTTGAAC 722
 A  A  A  E  N  F  T  V  L  I  K  N  N  I  R  F  P  A  L  N  195
TTCATCCGGAGGAACATCCTCCCAGGGATGAACGACGCCTACCTGAGGCGCTGCCACAGG 782
 F  I  R  R  N  I  L  P  G  M  N  D  A  Y  L  R  R  C  H  R  215
GGAAACGACTCGCTGTGTCCCATCTTCAGACTGGGAGACATCGTCCGAGAGGCCGGTGAG 842
 G  N  D  S  L  C  P  I  F  R  L  G  D  I  V  R  E  A  G  E  235
AAGTTCTCTGAAATGTCGGTAGAGGGGGGCGTCATCGGCATCCTGATCAAATGGGACTGT 902
 K  F  S  E  M  S  V  E  G  G  V  I  G  I  L  I  K  W  D  C  255
AACCTGGACCGGCTGATGCAGCGCTGCCTGCCCAGATACTCCTTCAGACGGCTGGACGAG 962
 N  L  D  R  L  M  Q  R  C  L  P  R  Y  S  F  R  R  L  D  E  275
AAAGAGAGCAACAAGACGCTGTACCCCGGCCTGAACTTCAGGTACGCAAAGTACAACACG 1022
 K  E  S  N  K  T  L  Y  P  G  L  N  F  R  Y  A  K  Y  N  T  295
GTGAACGGAGTGGAGGAGAGAACGCTGTACAAAGCATTCGGGATCAGGTTTGATGTCATG 1082
 V  N  G  V  E  E  R  T  L  Y  K  A  F  G  I  R  F  D  V  M  315
GTGTTCGGACAGGCGGGAAGGTTCAGCTTCATCCAGCTCATCATCTACATCGGATCAACG 1142
 V  F  G  Q  A  G  R  F  S  F  I  Q  L  I  I  Y  I  G  S  T  335
CTGTCGTACTACGCTCTGACAACCATATTGATCGACTGGCTGATTGGAACCAGCTGTTAC 1202
 L  S  Y  Y  A  L  T  T  I  L  I  D  W  L  I  G  T  S  C  Y  355
TCTGCAGAAGTCGGACAAAACTACTCAGATAAGAAAGTGGAGTCGGTCCGAGACAAACAG 1262
 S  A  E  V  G  Q  N  Y  S  D  K  K  V  E  S  V  R  D  K  Q  375
AAGTGCATCGTCTGTGTGTCCTACGTTGATGAGAACAACATTCGGCTGGTGAAGAGATCG 1322
 K  C  I  V  C  V  S  Y  V  D  E  N  N  I  R  L  V  K  R  S  395
CAGAAGAAAAGTTTACAAGACCTCAAGCCGACGTGTGTTCAGCCACGCAAGGAGGACGCA 1382
 Q  K  K  S  L  Q  D  L  K  P  T  C  V  Q  P  R  K  E  D  A  415
GGACACCTGAGAGCCGCTCTCTCTCTGCTGCAGCCGGGTGTCGGAGTGGATCACGACGCA 1442
 G  H  L  R  A  A  L  S  L  L  Q  P  G  V  G  V  D  H  D  A  435
GAGCCTCCCCTCGACCACAAACCCGACCCGAACCCCAAACCCCGCCGCCCGTCCTGGTGT 1502
 E  P  P  L  D  H  K  P  D  P  N  P  K  P  R  R  P  S  W  C  455
AAGTGCGACCGCTGCGCTCCCTCCCCTCTCCCCCAGGAGGAGCTGTGCTGCCGGCGGAGC 1562
 K  C  D  R  C  A  P  S  P  L  P  Q  E  E  L  C  C  R  R  S  475
GGCGGCGCCTGCATCACCTCGTCTCCTCTGTTTGAGCGACTGGTGCTGCGGCGCCCCCTG 1622
 G  G  A  C  I  T  S  S  P  L  F  E  R  L  V  L  R  R  P  L  495
CTGGAGGCCGTCCTCCTGTACCAGGACCCTCTGTCTCCACCTGCTGACCGAGGCCAGACC 1682
 L  E  A  V  L  L  Y  Q  D  P  L  S  P  P  A  D  R  G  Q  T  515
TCCGCCCTGCGCCACTGCGCCTACAGACAGTACATCAGCTGGAGGTTCGGGGTCCCGCCT 1742
 S  A  L  R  H  C  A  Y  R  Q  Y  I  S  W  R  F  G  V  P  P  535
GACGACACCCACCCCGCCGTGCCCAGCTGCTGCGTGTGGAGGGTCAGGCGAGACTACCCG 1802
 D  D  T  H  P  A  V  P  S  C  C  V  W  R  V  R  R  D  Y  P  555
AGCCCGGACGGACAGTACAGCGGCTTCGCACCTGCCAAGATGGCATCCATGCAGGACTGT 1862
 S  P  D  G  Q  Y  S  G  F  A  P  A  K  M  A  S  M  Q  D  C  575
GCTAACGGAGAGCTG**TGA**gaggagagtttccccattatagagcagggactcccccgctga 1922
 A  N  G  E  L  -  580
ctgacccactgacagtgttttatgttaaatctgtcttacaaagcaaaaaaaaaaaaaaaa 1982
aaaaaaaaaaaa 1994

**Supplementary Figure 1. cDNA and deduced amino acid sequence of *Lm*P2X2 (A), *Lm*P2X4 (B), *Lm*P2X5 (C) and *Lm*P2X7 (D).** The ORF is shown in upper case and the 5´-UTR and 3´-UTR sequences are in lower case. The translation initiation codon, stop codon and polyadenylation signal are shown in bold.

**A**

**
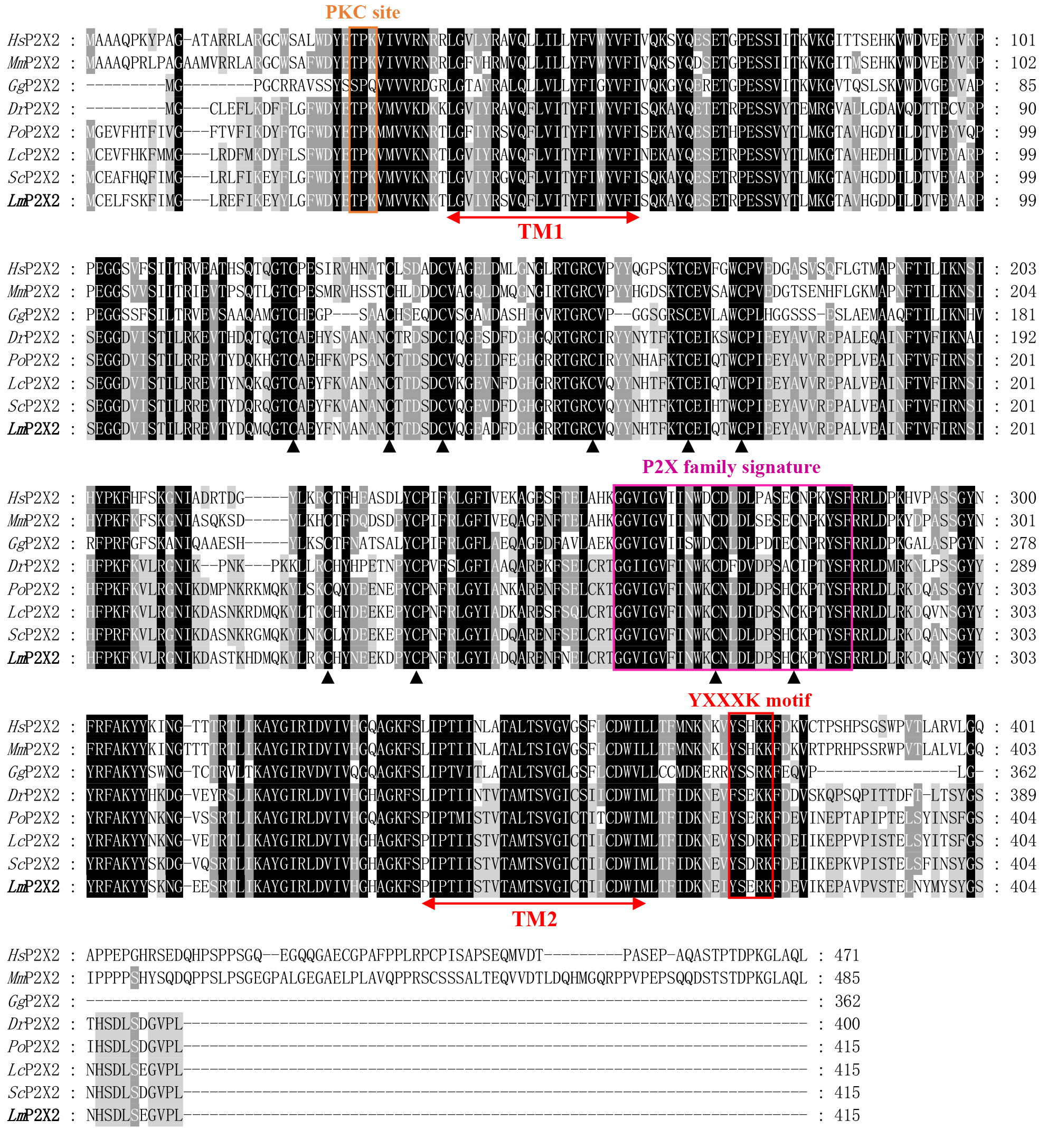
**

**B**

**
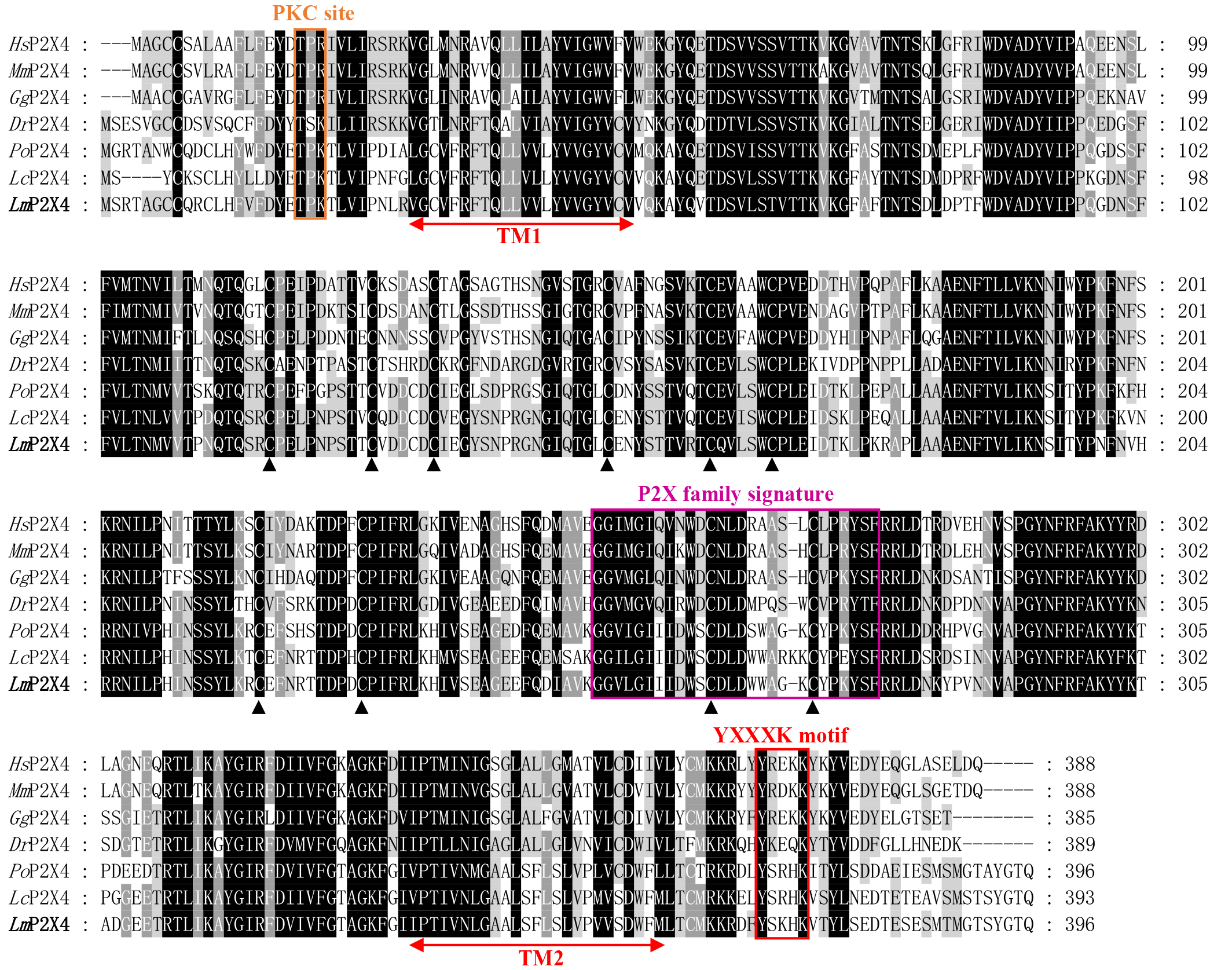
**

**C**

**
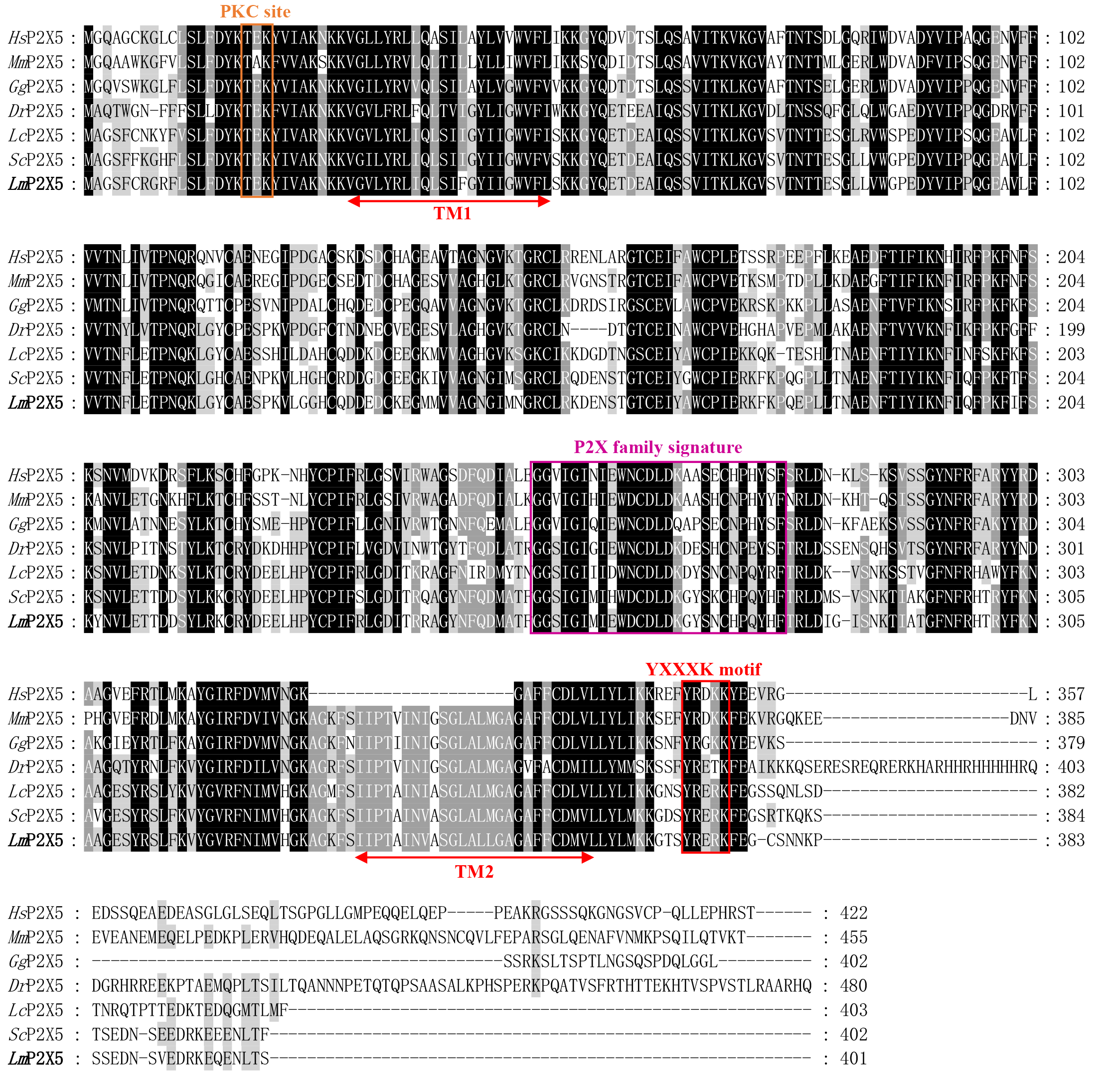
**

**D**

**
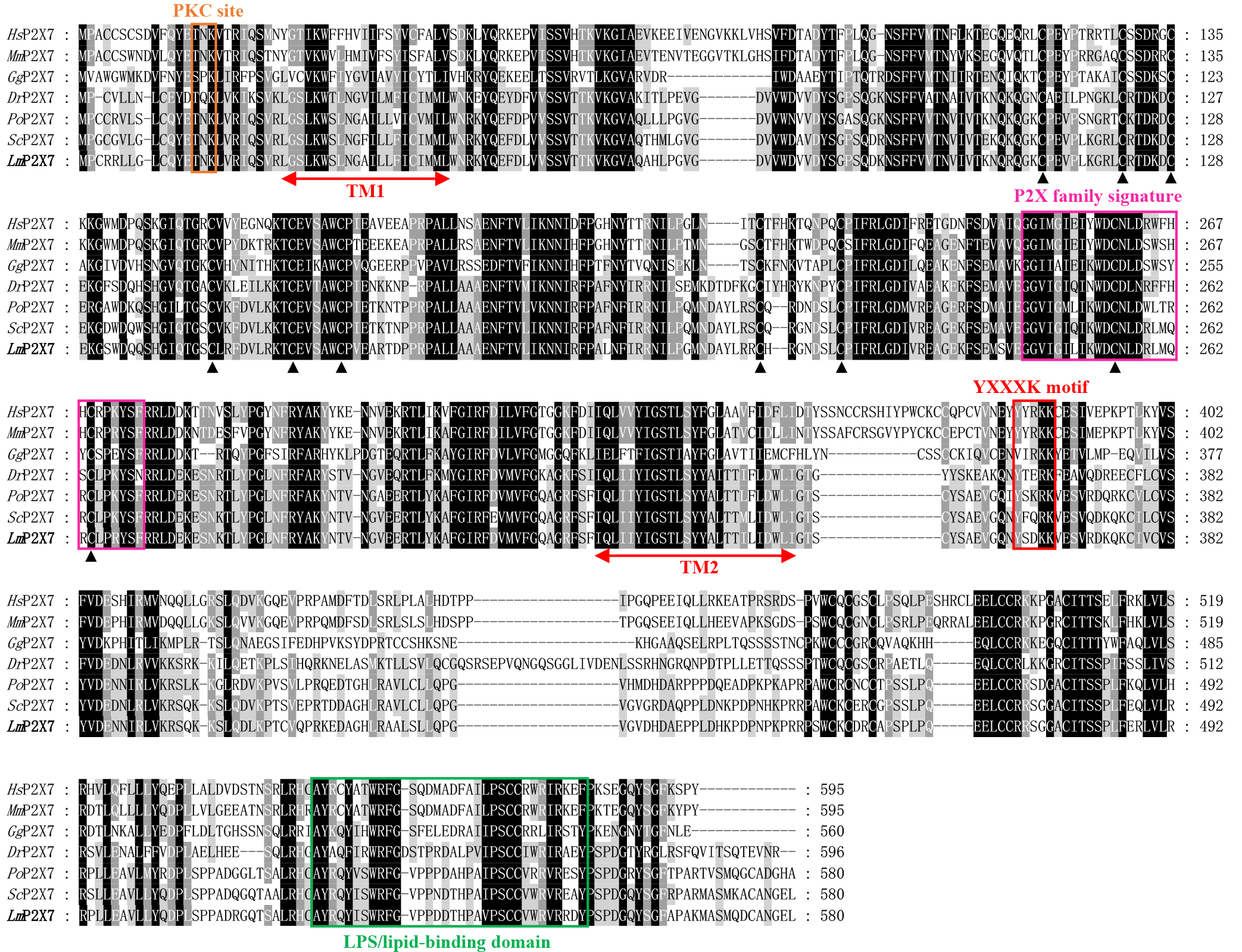
**

**Supplementary Figure 2. Multiple sequence alignment analysis of P2X2 (A), P2X3 (B), P2X4 (C), P2X5 (D) and P2X7 (E).** Two transmembrane domains (TM1-2) are marked below the alignment. Symbol (▲) indicates the conserved cysteine residues. The PKC site, P2X family signature motif, YXXXK motif and LPS/lipid-binding domain are boxed in orange, pink, red and green, respectively. The accession numbers of sequences are shown in Fig. 2. *Hs*: *Homo sapiens*, *Mm*: *Mus musculus*, *Gg*: *Gallus gallus*, *Dr*: *Danio rerio*, *Po*: *Paralichthys olivaceus*, *Lc*: *Larimichthys crocea*, *Sc*: *Siniperca chuatsi*, *Lm*, *Lateolabrax maculatus*

**
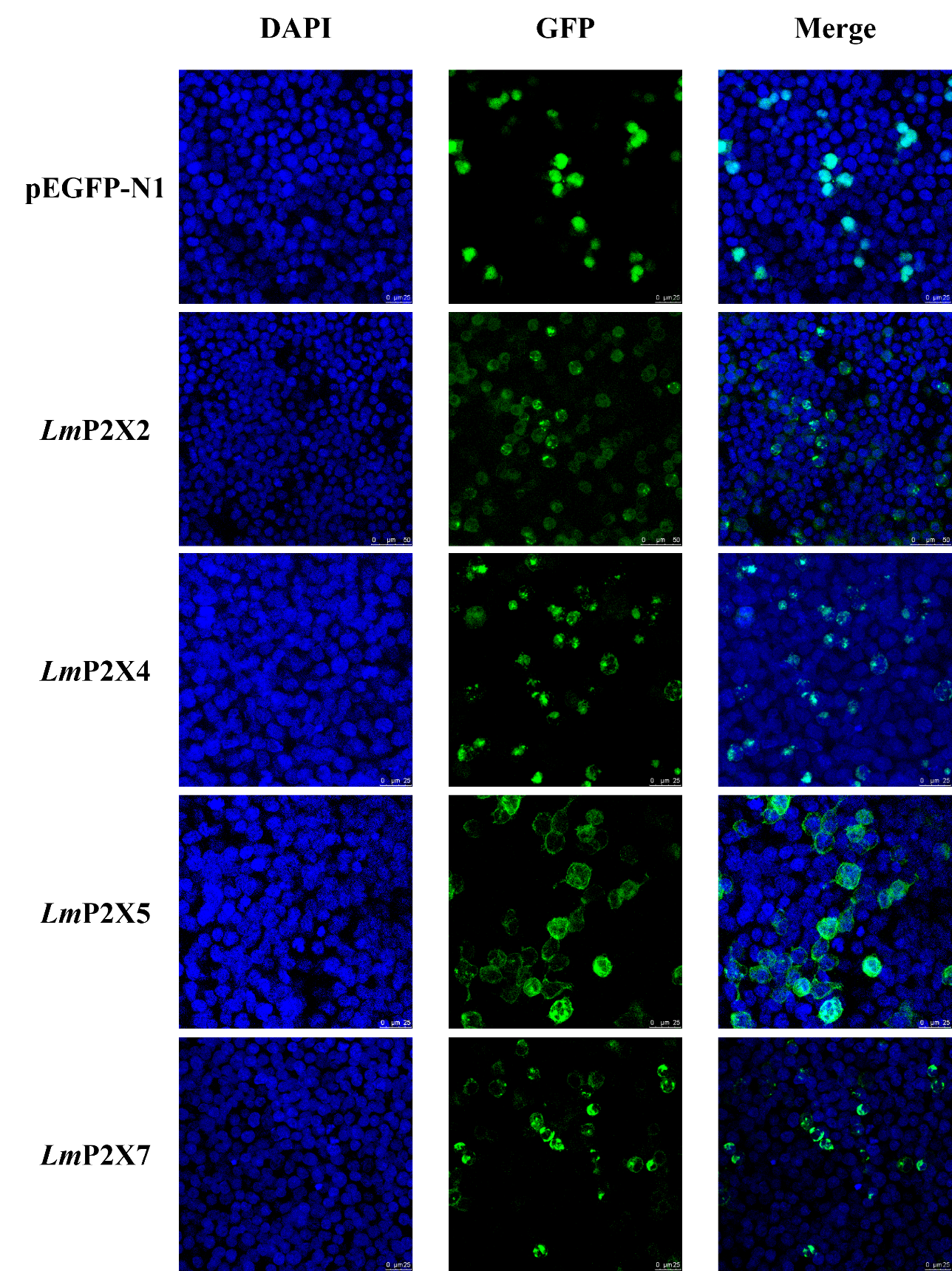
**

**Supplementary Figure 3. Subcellular localization of *Lm*P2Xs in HEK293 T cells.** HEK293 T cells were transfected with pEGFP-N1-*Lm*P2Xs recombinant plasmids or pEGFP-N1 empty plasmids. At 24 h post transfection, the cells were stained with DAPI and observed under a confocal microscope.
